# Clinical Diagnosis and Treatment of Iliopsoas-Related Groin Pain: A Systematic Review

**DOI:** 10.3390/jcm15155912

**Published:** 2026-07-29

**Authors:** Vandeputte Frans-Jozef, Sergooris Abner, Roose Stijn, Timmermans Annick, Corten Kristoff

**Affiliations:** 1 European Hip Center, Boskant 18, 2260 Westerlo, Belgium; frans.jozef.vandeputte@heuppraktijk.be (V.F.-J.); stijn.roose@heuppraktijk.be (R.S.);; 2 Hip Unit, Department of Orthopedics, Ziekenhuis Oost-Limburg, 3600 Genk, Belgium; 3 REVAL Rehabilitation Research, Faculty of Rehabilitation Sciences, Hasselt University, 3500 Diepenbeek, Belgium; annick.timmermans@uhasselt.be

**Keywords:** iliopsoas-related groin pain, psoas muscles, groin, tenotomy, arthroplasty, replacement, hip, sensitivity and specificity

## Abstract

**Background/Objectives**: Iliopsoas-related groin pain (IRGP) occurs in native hips and following hip surgery. However, diagnosis is challenging due to symptom overlap with other hip pathologies and limited imaging accuracy. Treatment ranges from conservative management to surgery. This systematic review evaluates the diagnostic accuracy of clinical tests and the effectiveness of conservative and surgical treatments for IRGP. **Methods**: This systematic review was conducted in accordance with the PRISMA 2020 guidelines and prospectively registered in PROSPERO. A comprehensive search of PubMed, Embase, and Web of Science was performed from database inception to 13 March 2026. Diagnostic accuracy studies evaluating clinical tests for IRGP against reference standards and therapeutic intervention studies assessing treatment effectiveness in individuals with IRGP were included. Risk of bias was assessed using QUADAS-3 for diagnostic studies and the MINORS tool for treatment studies. Due to heterogeneity in populations, interventions, outcomes, and study design, a narrative synthesis was performed. **Results**: Three diagnostic accuracy studies and 71 treatment studies were included. All three diagnostic studies used an image-guided injection as reference standard and were at high risk of bias. The hip–external rotation–flexion–ceiling (HEC) test showed the highest diagnostic accuracy (sensitivity 94%, specificity 88%), together with resisted seated hip flexion. However, all three diagnostic studies were at high risk of bias, so these estimates should be regarded as preliminary. Supine provocation tests showed high sensitivity but poor specificity. Conservative management achieved high success rates in idiopathic and athletic populations (77–100%) but lower and more variable effectiveness after total hip arthroplasty (THA) (16–50%). Image-guided injections produced short-term improvement in 50–70% of patients, with limited long-term effectiveness in mechanically driven cases after THA. In native hips, four matched comparative cohort studies indicated that arthroscopic iliopsoas release provided no clear benefit beyond treatment of concomitant intra-articular pathology and was associated with measurable iliopsoas atrophy and loss of hip flexion strength. After THA, both iliopsoas tenotomy and acetabular component revision improved pain and function, with outcomes strongly dependent on implant position and patient selection. Methodological quality of the treatment studies was generally low to moderate (MINORS 5–14/16 for non-comparative and 14–21/24 for comparative studies), while all three diagnostic studies were at high risk of bias on QUADAS-3. **Conclusions**: Current evidence highlights substantial diagnostic and therapeutic uncertainty in IRGP. Clinical tests lack sufficient standalone accuracy and should at least include the HEC test and resisted seated hip flexion. Treatment outcomes depend strongly on the underlying etiology, and the lack of an accurate diagnostic reference standard further complicates their interpretation. High-quality diagnostic studies and comparative intervention trials are needed to support evidence-based clinical pathways. **Registration**: PROSPERO CRD42025644593.

## 1. Introduction

Iliopsoas-related groin pain (IRGP) is increasingly recognized in both native hips and following hip surgery [1,2,3]. Anatomical predisposing factors include variants of the psoas valley [4] and anterior acetabular wall deficiency in the context of acetabular dysplasia [5]. After total hip arthroplasty (THA), several implant-related factors have been associated with iliopsoas impingement, including anterior cup overhang or oversizing, decreased cup anteversion, large femoral head size, prominent collared stem, and protruding acetabular screws or cement [6,7,8,9,10]. Patient-related factors such as female sex, younger age, and a history of spinal fusion further increase the risk of IRGP after direct anterior approach THA [9,11]. Its multifactorial etiology likely influences the substantial heterogeneity observed in clinical presentation, diagnostic findings, and response to treatment. The reported incidence of IRGP after THA varies between 2% and 11% depending on diagnostic criteria and surgical approach [8,9,11]. Given the steadily increasing number of THA procedures performed worldwide, even this relatively low incidence translates into a substantial and growing absolute burden of iliopsoas-related complications [12].

The iliopsoas is a key component of the peri-articular muscle envelope of the hip [13,14]. Although historically described as a primary hip flexor [15,16,17], more recent biomechanical work has demonstrated a broader functional role: the iliopsoas acts as an anterior femoral head stabilizer at 0–15° hip flexion, contributes to maintenance of an erect lumbar posture between 15–45°, and is recruited as a hip flexor only beyond 45° [17,18]. Electromyographic and biomechanical studies further support its role as an active stabilizer of the lumbar spine during gait and upright stance [19]. Disruption of this complex functional system may manifest as groin pain across a wide range of activities.

Clinically, IRGP is typically provoked by active or resisted hip flexion, rising from a seated position, stair climbing, or prolonged hip extension. In patients with internal snapping hip, tendon-snapping may be elicited during active hip flexion maneuvers [1]. Nevertheless, validated bedside tests with established diagnostic accuracy are scarce: pain during active hip flexion or passive hip extension is non-specific [1], and imaging modalities such as MRI and ultrasound have shown limited correlation with response to diagnostic injection [20]. Consequently, several authors have proposed diagnostic criteria centered on response to image-guided peritendinous anesthetic injection [21,22].

Treatment strategies for IRGP range from activity modification and physiotherapy to image-guided local injections, iliopsoas tenotomy, and acetabular component revision [1,2,23]. However, evidence-based and personalized diagnostic and rehabilitation pathways for IRGP—particularly after THA—are currently lacking, and previous systematic reviews have addressed either risk factors [9] or specific surgical techniques [24]. To date, however, no review has synthesized the diagnostic accuracy of clinical tests together with the full spectrum of conservative, injection-based, and surgical treatments across the different clinical contexts in which IRGP occurs, and no validated bedside test or standardized conservative protocol currently exists. This review is, therefore, the first to combine diagnostic accuracy and treatment evidence for IRGP and to stratify findings by clinical context (native hip, post-arthroscopy, post-THA), thereby reframing IRGP as a clinical umbrella term encompassing distinct pathophysiological entities and providing a mechanically driven framework to guide diagnosis, treatment selection, and future research.

The aim of this systematic review was, therefore, (1) to evaluate the diagnostic accuracy of clinical tests used to identify IRGP and (2) to assess the effectiveness of conservative, injection-based, and surgical treatments in reducing pain and improving functional outcomes in individuals with IRGP, both in native hips and after hip arthroplasty.

## 2. Materials and Methods

This systematic review was conducted in accordance with the Preferred Reporting Items for Systematic Reviews and Meta-Analyses (PRISMA) 2020 guidelines [25] and was prospectively registered in the PROSPERO database (CRD42025644593). The PRISMA 2020 checklist has been included in the Supplementary Materials (Table S1).

### 2.1. Eligibility Criteria

Studies were included based on eligibility criteria structured according to the PICOS framework. Eligibility criteria for each research question are summarized in Table 1.

Studies not involving human participants were excluded, as were case reports, narrative reviews, editorials, expert opinions, and conference abstracts without sufficient data.

Eligible study designs comprised diagnostic accuracy studies evaluating one or more clinical tests against a reference standard, and therapeutic studies (comparative studies, prospective and retrospective cohorts, and case series) reporting clinical outcomes after conservative, injection-based, or surgical treatment. The population, index tests, interventions, comparators or reference standards, and outcomes for each research question are detailed in Table 1.

### 2.2. Information Sources

A comprehensive literature search was conducted from database inception until 13 March 2026, in the following electronic databases: PubMed, Embase, and Web of Science. To ensure completeness, the reference lists of all included studies and relevant reviews were manually screened for additional eligible publications. The final search date for all sources was recorded, and no publication date restrictions were applied.

### 2.3. Search Strategy

The search strategy combined Medical Subject Headings (MeSH) and free-text keywords related to the population, interventions, reference standards or comparators, and outcomes, following the PICOS framework (Table 1). Search strategies were tailored to each database.

Full search strategies for PubMed are provided in Appendix A. Equivalent strategies were adapted for Embase and Web of Science. Searches were limited to studies involving human participants.

### 2.4. Selection Process

Identified records were all imported into Rayyan, a reference management software [26], and duplicates were removed. Two reviewers (FJVDP and AS) independently screened titles and abstracts for eligibility. Full texts of potentially eligible studies were retrieved and assessed independently by the same reviewers.

Disagreements at any stage of the selection process were resolved through discussion, and if necessary, by consultation with a third reviewer (SR). No automation tools were used in the screening process.

### 2.5. Data Collection Process

Data were extracted by one reviewer (AS) using a predefined data extraction form. Extracted data were checked for accuracy and completeness by a second reviewer (FJVDP). When required, corresponding authors were contacted to clarify missing or unclear information. No automated data extraction tools were used.

### 2.6. Data Items

#### 2.6.1. Outcomes

For diagnostic accuracy studies, data were extracted on sensitivity, specificity, likelihood ratios, and raw diagnostic data (true positives, false positives, true negatives, false negatives) where available. For each diagnostic study, we recorded how the index test cut-offs and the reference standard threshold for a positive response were defined, and whether these were pre-specified or data-driven.

For treatment studies, data were extracted on self-reported pain (e.g., visual analog scale (VAS), Numeric Rating Scale (NRS)), patient-reported outcome measures (PROMs) (e.g., Harris Hip Score (HHS), Hip disability and Osteoarthritis Outcome Score (HOOS), Oxford Hip Score (OHS), International Hip Outcome Tool (iHOT)), and performance-based outcomes. All reported time points were extracted when available.

#### 2.6.2. Other Variables

Additional data items included study design, sample size, patient characteristics, underlying etiology of IRGP (idiopathic, post-arthroscopy, post-THA), intervention characteristics, follow-up duration, and reported complications. No assumptions were made for missing data.

### 2.7. Study Risk of Bias Assessment

Risk of bias was assessed independently by two reviewers (FJVDP and AS), and any discrepancies were resolved through consensus.

For diagnostic accuracy studies, risk of bias and applicability were assessed using the QUADAS-3 tool [27]. Each domain (participants, index test, target condition, and analysis) was rated as “low”, “high”, or “insufficient information” according to predefined questions. Any domain judged as high risk resulted in an overall high risk of bias.

For treatment studies, methodological quality was assessed using the Methodological Index for Non-Randomized Studies (MINORS) tool [28]. Any discrepancies were resolved by consensus.

Because no meta-analysis was performed, formal statistical assessment of publication bias (e.g., funnel-plot asymmetry or Egger’s test) was not applicable; the potential for publication and positive outcome bias was, therefore, considered narratively, particularly in the surgical case series literature.

### 2.8. Effect Measures

For diagnostic accuracy outcomes, sensitivity, specificity, and likelihood ratios were reported. For treatment outcomes, effect measures included mean differences or changes from baseline in pain and functional scores, where available. Due to heterogeneity, standardized effect sizes were not consistently calculated.

### 2.9. Synthesis Methods

Studies were grouped according to research question (diagnosis vs. treatment), intervention type, and clinical context (native hip, post-arthroscopy, post-THA). Due to substantial clinical and methodological heterogeneity, a narrative synthesis was performed.

Data were tabulated to summarize study characteristics, outcomes, and risk of bias. No meta-analysis was conducted, as heterogeneity in populations, interventions, outcome measures, and study design precluded meaningful quantitative synthesis.

For the diagnostic question, only three studies were available, using different index-test definitions and reference standards and all at high risk of bias, so a bivariate or hierarchical summary ROC meta-analysis was not feasible. For the treatment question, the absence of randomized trials and the predominance of clinically heterogeneous, uncontrolled single-arm studies precluded valid pooling. A risk of bias-weighted narrative synthesis was, therefore, undertaken, as pre-specified in the PROSPERO protocol.

Subgroup analyses were performed descriptively based on etiology and treatment category. No sensitivity analyses were conducted.

## 3. Results

### 3.1. Study Selection and Design

**Figure 1:**
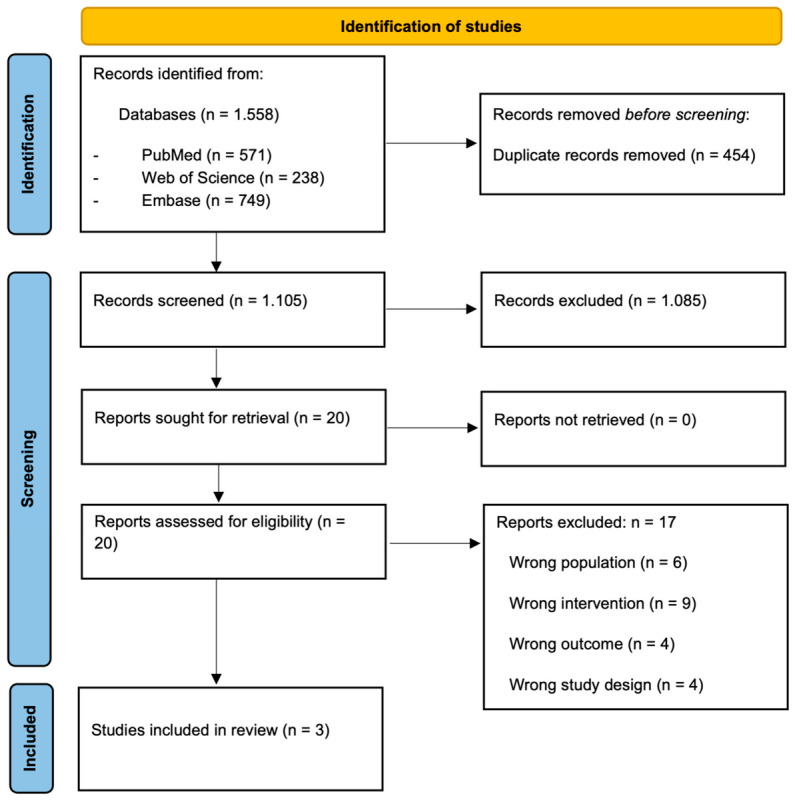
PRISMA 2020 flow diagram for diagnostic accuracy studies [25].

**Figure 2:**
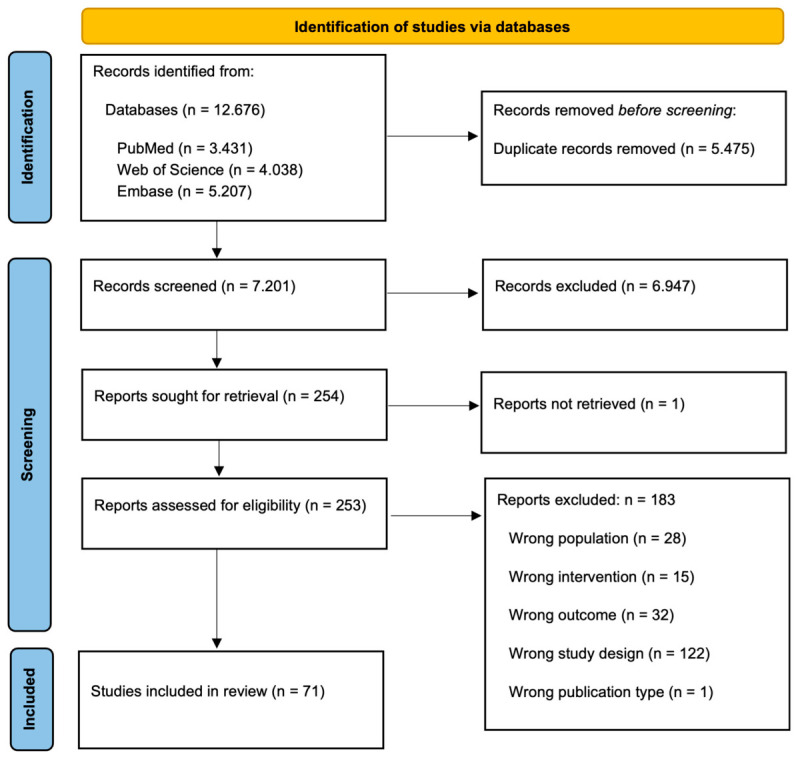
PRISMA 2020 flow diagram for intervention studies [25].

For this systematic review, three diagnostic accuracy studies addressing the clinical diagnosis of iliopsoas-related groin pain and 71 studies addressing treatment met the inclusion criteria (Figure 1 and Figure 2). All three diagnostic studies used a retrospective diagnostic cohort design and evaluated the performance of clinical tests against an injection-based reference standard. The 71 included treatment studies consisted predominantly of retrospective case series (n = 34), retrospective cohort studies (n = 30, of which four were comparative matched cohorts), and a limited number of prospective observational cohorts (n = 7). No randomized controlled trials were identified. Study characteristics and extracted outcomes for the diagnostic and treatment studies are summarized in Table 2 and Table 3, respectively. The included studies were published between 1999 and 2026.

### 3.2. Study Characteristics

The three included diagnostic studies comprised a total of 242 injections in 212 patients, with sample sizes of 44, 63, and 105 patients, respectively. Study populations were heterogeneous and included both individuals with native hips and individuals after THA or hip arthroscopy. Vandeputte et al. (2025) included 23 native hips (52%) and 21 after THA (48%) [29]. Haskel et al. (2021) included 22 native hips (35%), 34 after THA (54%), and seven after hip arthroscopy (11%) [20]. Johnstone et al. (2026) included 70 native hips (51.9%), 49 after hip arthroscopy (36.3%), nine after revision arthroscopy (6.7%), and seven after THA (5.2%) [30].

The included treatment studies represented a total of over 3000 hips, with sample sizes ranging from three to over 1300 patients. Considerable heterogeneity was observed across studies in terms of:

- **Etiology of IRGP:**
  ▪ Idiopathic iliopsoas tendinopathy/internal snapping hip;
  ▪ Iliopsoas pathology treated concomitantly during hip arthroscopy for femoroacetabular impingement (FAI) or dysplasia;
  ▪ Iliopsoas impingement following total hip arthroplasty (THA).
- **Interventions**: including conservative therapy, image-guided injections, arthroscopic/endoscopic iliopsoas procedures, and open iliopsoas tenotomy or revision arthroplasty.
- **Outcome measures**: with pain scores (VAS/NRS), hip-specific PROMs (mHHS, HHS, HOS, HOOS, OHS, iHOT-12), patient satisfaction, and return-to-sport outcomes most reported.

Follow-up duration ranged from 6 weeks to more than 10 years, with most surgical studies reporting outcomes between 24 and 60 months.

### 3.3. Diagnostic Accuracy of Clinical Assessment

#### 3.3.1. Reference Standard

All three included diagnostic accuracy studies (n = 212 patients; 242 injections) used an image-guided iliopsoas peritendinous injection with local anesthetic (with or without corticosteroid) as the reference standard. A positive diagnosis was defined by clinically meaningful pain relief following injection, although the threshold and timing of assessment for defining a positive response differed between studies. Vandeputte et al. graded each clinical test by test-induced VAS pain and derived the optimal cut-off data-driven using ROC analysis with the Youden J statistic within a single cohort, pre-specifying only the “good-test” criteria (pain reduction ≥ 4; AUC ≥ 0.80) [29]. In contrast, Haskel et al. [20] and Johnstone et al. [30] evaluated dichotomous physical examination and imaging findings without threshold optimization, with pre-specified reference standard thresholds (a ≥ 2-point NPRS reduction [MCID] and >50% pain reduction, respectively). All three studies were judged at high risk of bias on multiple QUADAS-3 domains (Table 4) because of heavy pre-selection of participants, an unblinded single examiner, and reliance on an imperfect reference standard. The proportion of patients with a positive reference standard (injection response) ranged from approximately 68% to 82% across the three studies. This variation, driven by differing response thresholds, timing of assessment, and participant pre-selection, produces a spectrum effect that tends to inflate sensitivity and limits the comparability of the reported accuracy estimates.

#### 3.3.2. Clinical Examination Tests

The best-supported finding is that seated hip flexion provocation tests outperform classical supine provocation tests. In Vandeputte et al. (2025), the HEC test (SN 94%, SP 88%, AUC 0.99, 95% CI 0.98–1.00), resisted hip flexion seated (SN 94%, SP 89%, AUC 0.96), and resisted hip flexion–external rotation seated (SN 96%, SP81%, AUC 0.98) all met the pre-specified “good test” criteria [29]. Johnstone et al. (2026) independently confirmed the high sensitivity of seated hip flexion testing (absolute SHF weakness SN 96.2%, PPV 77.1%), although specificity could not be calculated because of the pre-selected population [30]. Pain with seated hip flexion in Johnstone et al. (2026) (SN 76.1%, SP 22.2%) corroborated the same pattern [30].

Tests relying on supine provocation or palpation (straight-leg raise neutral, Thomas test, HEER, snapping hip test, tenderness medial to the joint) showed high sensitivity but consistently poor specificity in both Vandeputte et al. (2025) and Haskel et al. (2021), limiting their standalone diagnostic value [20,29]. Haskel et al. (2021) found that none of the four physical examination findings, ultrasound features (bursal distension, tendinosis), or MRI findings (bursal distension, tendon thickening, signal heterogeneity) were significantly associated with a positive injection response (all *p* > 0.05) [20]. Johnstone et al. (2026) reported that MRI abnormalities were present in only 18.1% of scanned hips, yielding a SN of 19.2% and an SP of 85.0%, and confirming that MRI is insensitive but specific for iliopsoas pathology [30].

Given the small evidence base (three studies, all at high risk of bias) and the absence of a validated gold standard, no single clinical test can yet be recommended as a standalone diagnostic test. Clinical tests should at least include the HEC test and resisted seated hip flexion.

### 3.4. Treatment Effects by Intervention Category and Etiology

Because IRGP arises in distinct clinical contexts—idiopathic tendinopathy or internal snapping hip in the native hip, iliopsoas pathology addressed during hip arthroscopy for FAI or dysplasia, and mechanically driven impingement after THA—that differ in mechanism, diagnostic pathway, and treatment implications, treatment outcomes are presented within these contexts and by intervention category below.

#### 3.4.1. Conservative Treatment

Conservative management was evaluated in six studies (n = 102 in conservative treatment arms): three small retrospective case series in native hips [31,32,33] and three retrospective multi-arm comparative cohort studies that included a conservative arm for iliopsoas impingement after THA [22,92,93].

In younger and athletic populations, targeted physiotherapy with iliopsoas stretching, hip rotator and pelvic strengthening, motor control, and activity modification produced high rates of symptom resolution. Johnston et al. (1999) reported that 77% of patients returned to full activity following a structured home-based rehabilitation program [31], and Laible et al. (2013) reported 100% clinical success in a cohort of dancers without injections or surgery [32]. These were, however, small single-center case series at high risk of bias (MINORS 8–12/16) and should be regarded as hypothesis-generating rather than definitive.

In individuals with iliopsoas impingement after THA, the strongest available evidence comes from three retrospective multi-arm comparative cohorts [22,92,93]. These studies converge on the same conclusion: conservative treatment provides meaningful benefit in only 48–50% of patients [92,93]; the remaining patients require injection or surgical intervention. Moreover, conservative treatment alone resulted in only marginal improvement on HHS from 57 to 58 [22].

In summary, conservative treatment is effective in idiopathic iliopsoas pathology but demonstrates limited and inconsistent benefit in mechanically driven iliopsoas impingement, particularly after THA.

#### 3.4.2. Image-Guided Iliopsoas Injections

Image-guided injections were evaluated in eight studies (n = 422) as both a diagnostic and therapeutic intervention: five in idiopathic or mixed populations [21,34,35,36,37] and three in individuals after THA [66,89,94]. All injection studies were non-comparative retrospective designs (MINORS 9–16/16); no studies compared injections to no injections.

In idiopathic iliopsoas tendinopathy, injections produced short-term pain reduction and functional improvement in a substantial proportion of patients. Agten et al. (2015) reported clinically relevant improvement in 49% at one month, with significant NRS reduction (5.9 to 3.5, *p* < 0.001), and noted no difference between individuals with and without THA [34]. Han et al. (2019), the largest cohort (n = 178), demonstrated significant improvements across all HOOS subscales irrespective of concomitant intra-articular hip pathology [35]. Blankenbaker et al. (2006) achieved the highest MINORS score of the injection studies and reported sustained pain relief at 4 months in 89% (16/18) of individuals managed with a corticosteroid bursal injection only [36].

In individuals after THA, injections produced initial relief in approximately 60–70% of patients, but recurrence was common. Howell et al. (2021) reported that 44% of initial responders later required surgical intervention [94], and Nunley et al. (2009) found that 22% of injected patients required additional surgery within a mean of 44 months [66].

In summary, iliopsoas injections provide diagnostic confirmation and short-term symptom relief in idiopathic cases and after THA. Long-term efficacy as a standalone treatment is, however, limited in individuals after THA, where recurrence and progression to surgery are common.

#### 3.4.3. Arthroscopic Procedures in Native Hips

Arthroscopic iliopsoas tenotomy or iliopsoas fractional lengthening (IFL) was investigated in 17 studies (n = 969), predominantly in patients undergoing hip arthroscopy for internal snapping hip with concomitant FAI or labral pathology.

The strongest evidence comes from four retrospective matched comparative cohort studies [38,39,40,41] (n = 501 IFL vs. 476 matched controls). Across these four studies, both the IFL and the control groups showed significant improvement in all PROMs, but no clear additional benefit was attributable to iliopsoas lengthening itself. In Maldonado et al. (2018), MCID and PASS rates were essentially identical between IFL and control (MCID 75.7% vs. 74.0%, *p* = 0.71; PASS 77.6% vs. 75.3%, *p* = 0.57) [38], and Jimenez et al. (2022) and Perets et al. (2018) reached the same conclusion [39,40]. Maldonado et al. (2021) confirmed comparable PROM improvement and MCID achievement in patients with borderline acetabular dysplasia after the combined FLIP procedure [41].

Three additional comparative cohort designs add complementary evidence. Brandenburg et al. (2016) (n = 18 release vs. 18 controls) found a 25% smaller iliopsoas muscle volume and 19% lower seated hip flexion strength on the operated side at 21 months (*p* < 0.001) [51]. Meghpara et al. (2020) evaluated 38 hips with iliopsoas impingement-associated labral pathology without painful snapping and compared them to 87 hips with painful snapping treated with IFL. Both subgroups demonstrated comparable improvements in PROMs [47]. Matsuda et al. (2021) compared 16 hips treated with iliopsoas tenotomy to 76 hips managed without tenotomy, alongside 1301 control hips, and reported that only 25% of hips undergoing tenotomy achieved normal function, compared with 41–49% in the non-tenotomy groups, suggesting inferior rather than equivalent functional recovery following iliopsoas release [50]. Smaller single-arm case series [43,44,45,46,48,49,52,53] are consistent with the symptomatic benefit reported in the comparative studies but do not allow attribution of effect to the iliopsoas-specific component.

In summary, arthroscopic iliopsoas procedures produce reliable symptomatic improvement and snapping resolution (80–95% across comparative studies). However, the four matched comparative cohort studies consistently indicate that they may not add measurable benefit beyond treatment of the concomitant intra-articular pathology, while the three additional comparative cohort designs indicate a quantifiable cost in iliopsoas volume, seated hip flexion strength, and possibly functional recovery.

#### 3.4.4. Endoscopic Procedures in Native Hips

Endoscopic iliopsoas tendon release was evaluated in four small non-comparative retrospective case series (n = 60) [54,55,56,57]. MINORS scores were at the lower end of the methodological quality spectrum (10-14/16), and no comparative data are available in this setting.

These studies report short-term improvement in pain and function, with no clear differences between a lesser trochanter and a transcapsular release approach. Loppini et al. (2026) was the only study with long-term (>10 years) follow-up and reported sustained improvement in mHHS (63.7 to 88.3) and HOOS (71.5 to 86.7), although 15% of individuals had persistent hip flexion weakness at last follow-up [57].

In summary, the endoscopic evidence is confined to small, heterogeneous non-comparative case series; it indicates reliable short-term pain and functional improvement, but the durability of benefit, the optimal level of release (lesser trochanter vs. transcapsular), and the risk of persistent hip flexion weakness remain uncertain.

#### 3.4.5. Open Surgical Procedures in Native Hips

Open iliopsoas tenotomy or IFL was evaluated in four retrospective studies (n = 136, 147 hips) [58,59,60,61]. One study had an internal comparator group, comparing tenotomy with injection [58], while the remaining three were non-comparative case series or chart reviews (MINORS 8–10/16).

Across these series, open tenotomy and IFL produced generally favorable outcomes, with high rates of pain relief and patient satisfaction reported across retrospective studies [58,59,60,61]. However, the results are more variable in the largest cohort, with reported complications including persistent pain, sensory deficits, and recurrent snapping [59]. The single comparative study suggested that open tenotomy was not superior to less invasive interventions, such as image-guided injections [58].

In summary, the open surgery evidence in native hips is limited to a few small retrospective series with variable outcomes and notable complications in the largest cohort; it shows no clear advantage over less invasive options, and open techniques are, therefore, best reserved for selected cases in which arthroscopic or endoscopic approaches are unsuitable.

#### 3.4.6. Iliopsoas Impingement After Total Hip Arthroplasty

The largest body of evidence addressed iliopsoas impingement following THA (n = 1052). Studies were distributed across endoscopic tenotomy (11 studies, n = 444), arthroscopic tenotomy/release (11 studies, n = 214), open tenotomy (four studies, n = 45), acetabular cup revision (five studies, n = 146), anterior capsule-thickening/wall reconstruction techniques (three studies, n = 26), targeted injection (three studies, n = 72), and multi-arm stepwise protocols (four studies, n = 105). Importantly, no matched comparative cohort studies are available in the post-THA setting. The strongest designs are five retrospective multi-arm comparative cohorts [8,22,92,93,95], without 1:1 demographic matching.

Endoscopic or arthroscopic iliopsoas tenotomy consistently produced significant pain reduction and functional improvement in individuals with iliopsoas cup impingement. Bell et al. (2019) reported pain resolution in 93.3% of patients at a mean follow-up of 5.5 months [63]; Pate et al. (2025) reported 80% pain-free individuals at 3.8 years [96]; Viamont-Guerra et al. (2021) reported improvement in mHHS (57.7 to 83.2) and OHS (29.4 to 50.5) at 31 months [68]; Erard et al. (2024) reported MCID achievement in 92.5% at 68.8 months [85]; and Lambrey et al. (2023) reported improvement in OHS (21.8 to 40.7) at 5 years with 80% satisfaction [91]. Several studies, however, documented a measurable strength deficit after tenotomy. Finsterwald et al. (2024) found a 20% decrement in peak isometric hip flexion strength on the operated side at 2 years, and Portet et al. (2024) reported 37–60% reductions in seated and supine flexion strength [69,72].

Comparative data on open versus arthroscopic technique are limited to Holliday et al. (2025) (n = 52, MINORS 16/24). Arthroscopic IFL trended toward higher satisfaction (7.9 vs. 6.0), greater groin pain improvement (86% vs. 45%, *p* = 0.065), lower VAS at rest (1.1 vs. 3.3, *p* = 0.009), and a lower revision THA rate (8.3% vs. 25%, *p* = 0.10) than open tenotomy [95].

Acetabular cup revision produced substantial clinical improvement in cases with pronounced cup prominence or malposition. Batailler et al. (2017) (n = 46) reported 85% satisfaction and improvement in OHS (19.7 to 43) at 21 months, but with 6.5% complications and 8.7% recurrent groin pain [62]. Martinot et al. (2025) (n = 55) reported MCID achievement in 87% and PASS in 67% at 3 years, but major complications in 10.9%, underlining the higher morbidity profile compared with tenotomy [80].

The three multi-arm retrospective comparative cohorts that directly compared interventions in the same study [8,22,92] consistently favor cup revision over tenotomy when cup malposition or anterior prominence is the mechanical driver, while tenotomy is favored for impingement without component malposition. Carbonell-Rosell et al. (2022) proposed an axial cup overhang cut-off of 10 mm to predict poor outcome after iliopsoas tenotomy alone [93].

Alternative tendon-sparing techniques were described in two small series. Benad et al. (2015) (n = 5, MINORS 5/16) reported short-term pain relief after anterior capsule-thickening with a Vicryl™ mesh via an abridged direct anterior approach [64], but a longer-term evaluation by the same group (n = 14) reported only 50% satisfaction at 4 years and a substantial proportion requiring secondary surgery [90]. Nogier et al. (2024) (n = 7) described an autologous iliac crest anterior wall reconstruction with concomitant cup revision and reported promising 5-year outcomes (MRC 3.9 to 4.6; satisfaction 8.3/10) but with very small numbers [5].

In summary, both iliopsoas tenotomy and acetabular cup revision are effective for iliopsoas impingement after THA, with high rates of symptomatic improvement across all series. The three best-quality multi-arm comparative cohorts [8,22,92] support a stepwise mechanically driven algorithm: tenotomy as first-line surgical treatment for impingement without component malposition, and cup revision when there is meaningful anterior cup overhang or malposition. Strength deficit after tenotomy is consistently quantifiable (20–60% reduction) and should be discussed with patients pre-operatively.

#### 3.4.7. Comparative Effectiveness

Direct comparisons between surgical approaches remain scarce. In native hips, arthroscopic fractional lengthening, endoscopic release, and open tenotomy produced broadly comparable short-term symptomatic improvement, with no high-quality head-to-head data; the only comparative study suggested possibly inferior functional recovery after tenotomy [50]. After THA, the single direct comparison of open versus arthroscopic technique favored the arthroscopic approach across satisfaction, groin pain improvement, resting VAS, and revision THA rate [95]; all other cross-approach comparisons remain indirect.

### 3.5. Risk of Bias Within Studies

All three included diagnostic studies were judged at high risk of bias on multiple QUADAS-3 domains (Table 4). The main sources of bias were participant pre-selection, absence of examiner blinding, and reliance on an imperfect, subjective injection-based reference standard.

Methodological quality of the treatment studies, assessed with the MINORS tool, was generally low to moderate (Table 5). Non-comparative studies scored 5–14/16 (median 10) and comparative studies scored 14–21/24 (median 17). Common limitations included retrospective design, absence of a priori sample size calculations, lack of blinded outcome assessment, and incomplete reporting of losses to follow-up.

Only four of the 71 included treatment studies were retrospective matched comparative cohort studies [38,39,40,41], all of which evaluated arthroscopic iliopsoas fractional lengthening against demographically matched controls without iliopsoas surgery. These four studies were given the greatest evidentiary weight in the narrative synthesis. Three additional comparative cohort designs [47,50,51] provided complementary evidence on biomechanical sequelae and functional outcomes after arthroscopic iliopsoas surgery. In the post-THA setting, no matched comparative cohorts were available; the strongest designs were five multi-arm retrospective cohorts [8,22,92,93,95] that compared interventions head-to-head within the same cohort. Baseline equivalence was often incompletely demonstrated even in these comparative designs, indicating residual confounding.

A formal assessment of risk of bias due to missing results could not be performed because of heterogeneity and the absence of a quantitative synthesis.

## 4. Discussion

### 4.1. Summary of Findings

This systematic review summarized the current evidence on the clinical diagnosis and treatment of IRGP. First, the evidence on the diagnostic accuracy of clinical tests is sparse and of low quality: only three studies, all retrospective and at high risk of bias, have evaluated clinical tests against an imperfect injection-based reference standard [20,29,30]. Within this limited evidence base, the hip–external rotation–flexion–ceiling (HEC) test showed the most favorable accuracy, but this rests on a single cohort and should be regarded as preliminary and hypothesis-generating. Second, conservative management strategies are poorly described and insufficiently standardized. Moreover, the treatment literature is dominated by surgical case series, with very few comparative studies and no randomized controlled trials. Finally, emerging evidence suggests that patient selection and component positioning strongly influence treatment outcomes, particularly for surgical interventions, such as iliopsoas tenotomy [70,93,97]. Therefore, IRGP should be considered a clinical umbrella term encompassing distinct pathophysiological entities. It should be interpreted and managed within its specific clinical context (e.g., idiopathic tendinopathy, post-arthroscopy pathology, and mechanically driven impingement after total hip arthroplasty).

Compared with previous reviews, our findings both confirm and extend the literature. Earlier reviews addressed only isolated aspects of IRGP: Minelli et al. [9] investigated rerisk factors for iliopsoas impingement after THA, Coulomb et al. [24] appraised the technique of arthroscopic iliopsoas tenotomy, and the scoping and narrative reviews of Tramer et al. [2] and Younis et al. [23] summarized anatomy and management. Most directly comparable is the recent systematic review and meta-analysis of Razick et al. [98], which pooled non-operative versus operative outcomes for iliopsoas impingement after THA. We confirm their results of favorable short-term surgical outcomes and are concordant with Razick et al. for the post-THA setting: conservative care frequently failed (persistent symptoms in 53.6% and progression to surgery in 16.4), both iliopsoas tenotomy and acetabular revision were effective, and revision carried a markedly higher complication rate (15.7% vs. 2.3% for tenotomy). Our review adds a formal appraisal of the diagnostic accuracy of clinical tests and a cross-setting, mechanically driven synthesis spanning native hips, post-arthroscopy, and post-THA, whereas Razick et al. remained limited to retrospective, non-randomized, heterogeneous post-THA data. Our more cautious overall interpretation reflects our structured QUADAS-3 and MINORS risk of bias appraisal and the absence of controlled comparative data.

### 4.2. Diagnostic Considerations

Accurate diagnosis is a critical first step in the management of IRGP yet remains one of the most challenging aspects. Clinical presentation often overlaps with femoroacetabular impingement, labral pathology, rectus femoris tendinopathy, and lumbar spine-related pain, while imaging findings frequently show poor correlation with symptoms [20]. Despite the clinical relevance of this condition, only three studies have formally evaluated the diagnostic accuracy of clinical tests against a reference standard [20,29,30]. All three were retrospective, at high risk of bias, and relied on an imperfect injection-based reference standard, so their accuracy estimates must be interpreted with considerable caution.

All three studies demonstrated limited diagnostic performance for commonly used clinical maneuvers, underscoring that no single test can currently be used in isolation. Importantly, however, the HEC test emerged as the most promising clinical test, showing the highest sensitivity and specificity compared with diagnostic injection or imaging-based reference standards [29]. In contrast, imaging modalities such as MRI and ultrasound appear insufficiently specific, reinforcing the frequent reliance on diagnostic injections as a reference standard in both diagnostic and therapeutic algorithms [20]. The apparent superiority of the HEC test, however, rests on a single cohort at high risk of bias and should be regarded as preliminary and hypothesis-generating. On current evidence, no single clinical test can be recommended for standalone diagnostic use.

### 4.3. Treatment Strategies

The treatment literature is characterized by a predominance of surgical studies, with limited high-quality evidence supporting non-operative interventions. Importantly, the lack of an accurate clinical diagnosis also complicates the reporting of treatment results. Most included studies focused on arthroscopic or endoscopic iliopsoas release or tenotomy, often in the context of femoroacetabular impingement or after THA. In contrast, conservative management strategies—including physiotherapy, activity modification, and exercise-based rehabilitation—were poorly described, highly variable, and rarely evaluated using standardized protocols.

Notably, there are no randomized controlled trials and only a small number of comparative studies evaluating treatment effectiveness. One of the few comparative studies is that of Maldonado et al., who compared outcomes of patients with iliopsoas tendinopathy undergoing hip arthroscopy with or without IFL. Their findings demonstrated comparable improvements in patient-reported outcomes between groups, suggesting that iliopsoas release may not be necessary in all patients and highlighting the importance of appropriate patient selection [38]. This study is particularly relevant, as it suggests that groin pain symptoms may, in some cases, resolve when underlying intra-articular pathology is adequately addressed.

### 4.4. Prognosis and Patient Selection

Although it was not an aim of this systematic review, an important emerging theme across studies is that anatomical and biomechanical factors influence prognosis. Fabricant et al. demonstrated that increased femoral anteversion was associated with inferior postoperative outcomes after iliopsoas lengthening, suggesting that altered femoral version may predispose patients to persistent symptoms or functional deficits [42]. Similarly, Carbonell-Rosell et al. (2022) identified an overall axial cup overhang threshold of 10 mm, which accurately discriminated between patients who did and did not improve with surgical treatment [93]. Nearly half of patients improved with conservative management, while those with greater axial and sagittal overhang were more likely to require tenotomy. Importantly, even among surgically treated patients, excessive overhang was associated with poorer outcomes. During primary THA, bony coverage of the cup must be checked, especially in the psoas groove, between the anterior inferior iliac spine and pubic eminence.

From a preventive standpoint, avoiding anterior cup overhang and oversizing, ensuring adequate anterior bony coverage of the cup, restoring appropriate cup anteversion, and avoiding prominent screws, collars, or protruding cement during primary THA represent practical intra-operative targets to reduce the risk of subsequent iliopsoas impingement [93].

These clinical observations are supported by emerging biomechanical evidence [99,100]. Cadaveric studies have demonstrated that anterior cup protrusion, increased stem offset, and unfavorable stem version substantially increase localized surface pressure on the iliopsoas muscle, particularly during hip extension [100]. Tamaki et al. (2023) showed that even moderate anterior cup protrusion resulted in marked increases in peak iliopsoas surface pressure, providing a mechanistic explanation for persistent symptoms in patients with mechanically driven pathology despite surgical intervention [100]. Moreover, experimental data suggest that the anterior hip capsule may act as a protective buffer between the iliopsoas tendon and the acetabular component, with capsular preservation or thickening reducing localized pressure on the iliopsoas compared with capsular resection [99]. However, this is currently not supported by clinical studies in individuals undergoing THA, where no significant association between capsular status and iliopsoas-related symptoms has been observed [101,102].

Together, these findings underscore that IRGP is not a homogeneous clinical entity. Mechanically driven pathology following THA appears to represent a distinct subgroup in which treatment success depends not only on the chosen surgical technique but also on underlying anatomy, implant positioning, and peri-articular soft tissue interactions. This highlights the importance of careful patient selection and individualized treatment strategies when considering iliopsoas tenotomy or alternative surgical approaches.

### 4.5. Risks of Surgical Intervention

While surgical intervention can provide reliable symptom relief in selected patients, potential risks must be carefully considered. Several studies have reported concerns regarding iliopsoas muscle atrophy, loss of hip flexion strength, and potential instability following tenotomy or fractional lengthening [18,42,51,98,103]. Although comparative data suggest that functional outcomes may remain acceptable in the short to mid-term, long-term effects on strength and dynamic hip stability remain insufficiently studied. These risks further emphasize the need for precise diagnosis and patient selection.

### 4.6. Limitations and Future Directions

The findings of this review must be interpreted considering several limitations, including heterogeneity of study designs, interventions, outcome measures, and diagnostic criteria, as well as the predominance of non-randomized studies. Future research should prioritize high-quality diagnostic accuracy studies, development of standardized conservative treatment protocols, and comparative trials evaluating both operative and non-operative strategies. Improved understanding of anatomical and biomechanical modifiers may ultimately allow for more personalized treatment algorithms. In line with the pre-specified protocol, a meta-analysis was not performed because the diagnostic and treatment evidence was too sparse and heterogeneous for valid quantitative pooling. For the same reason, publication bias could not be assessed statistically; given the predominance of retrospective, single-arm surgical case series reporting favorable outcomes, a high likelihood of publication and positive outcome bias should be assumed, and the reported treatment success rates should be interpreted with corresponding caution.

## 5. Conclusions

Current evidence highlights substantial diagnostic and therapeutic uncertainty in IRGP. Clinical tests lack sufficient standalone accuracy and should at least include the HEC test and resisted seated hip flexion. Treatment outcomes depend strongly on the underlying etiology, and the lack of an accurate diagnostic reference standard further complicates their interpretation. High-quality diagnostic studies and comparative intervention trials are needed to support evidence-based clinical pathways.

## Figures and Tables

**Table 1:** Inclusion criteria per research question according to PICO framework.

	Diagnostic Accuracy	Treatment Effectiveness
***Population***	Individuals with suspected IRGP.	Individuals with a clinical diagnosis of IRGP.
***Intervention***	Clinical tests used to identify IRGP, including physical examination maneuvers or other bedside tests.	Any therapeutic intervention aimed at reducing pain or improving function in individuals with IRGP. Examples include but are not limited to surgical procedures, injection therapy, exercise therapy, medication, passive modalities, and shock wave therapy.
***Control***	A reference standard such as magnetic resonance imaging (MRI), diagnostic infiltration, ultrasound, or surgical findings.	No specific comparator will be required; studies with single-arm designs or comparative interventions will be included.
***Outcome***	Outcomes including sensitivity, specificity, likelihood ratios (LRs), true positives (TPs), false positives (FPs), false negatives (FNs), and true negatives (TNs).	Outcomes including self-reported pain (e.g., VAS, NRS), patient-reported outcome measures (e.g., HHS, MHHS, HOOS, OHS, iHOT), disability, and performance-based measures.
***Study design***	Diagnostic accuracy studies (e.g., non-experimental cross-sectional design).	Randomized controlled trials, as well as non-randomized intervention studies, will be eligible.

Abbreviations: IRGP = iliopsoas-related groin pain, VAS = visual analog scale, NRS = Numeric Rating Scale, HHS = Harris Hip Score, MHHS = Modified Harris Hip Score, HOOS = Hip disability and Osteoarthritis Outcome Score, OHS = Oxford Hip Score, iHOT = International Hip Outcome Tool.

**Table 2:** Characteristics and diagnostic accuracy of the included diagnostic accuracy studies on iliopsoas-related groin pain.

Study (Year)	Design	Participants	Index Test(s)	Reference Standard	Outcome Measures	Main Results
Vandeputte et al. (2025) [29]	Retrospective diagnostic accuracy study	44 patients (29 F); mean age 48 ± 15 yr. 23 native hips, 21 post-THA. Reference-positive: 82% (36/44).	11 clinical tests: HEC test; SLR neutral; SLR external rotation; FADIR; snapping hip; scour test; Thomas test; HEER; resisted hip flexion (seated); resisted hip flexion–external rotation (seated); palpation medial to the hip.	Fluoroscopy-guided peritendinous iliopsoas injection (medial to the inferior anterior iliac spine, lateral to the pubic eminence). Positive response: patient-rated improvement in characteristic groin pain at 2 h (binary).	SN, SP, AUC, and optimal VAS reduction cut-off (ROC + Youden J).	HEC: SN 94%/SP 88%/AUC 0.99 (cut-off ≥ 5) Resisted hip flexion (seated): SN 94%/SP 89%/AUC 0.96 (cut-off ≥ 5) Resisted hip flexion–ER (seated): SN 96%/SP 81%/AUC 0.98 (cut-off ≥ 4) SLR external rotation: SN 78%/SP 80%/AUC 0.88 (cut-off ≥ 6) SLR neutral: SN 80%/SP 50%/AUC 0.72 (cut-off ≥ 5) Scour test: SN 79%/SP 77%/AUC 0.80 (cut-off ≥ 5) Tenderness medial to joint: SN 100%/SP 63%/AUC 0.87 (cut-off ≥ 4) FADIR: SN 100%/SP 78%/AUC 0.84 (cut-off ≥ 4) Thomas: SN 100%/SP 44%/AUC 0.70 (cut-off ≥ 3) HEER: SN 100%/SP 31%/AUC 0.65 (cut-off ≥ 3) Snapping hip (n = 9): SN 100%/SP 33%/AUC 0.72 (cut-off ≥ 5)
Haskel et al. (2021) [20]	Retrospective diagnostic accuracy study	63 patients (36 F); mean age 52.3 ± 17.3 yr. 54% post-THA, 35% no prior surgery, 11% post hip arthroscopy. Reference-positive: 68% (43/63).	PE: groin pain, snapping hip, pain with resisted SLR, weakness with resisted SLR. Ultrasound: bursal distension, tendinosis. MRI: bursal distension, tendon thickening, signal heterogeneity, tendon tear.	Ultrasound-guided peritendinous corticosteroid + lidocaine injection. Positive response: immediate NPRS reduction > 2 (MCID) and/or documented clinical improvement at follow-up.	SN, SP, PPV, and NPV for each PE manoeuvre and for US/MRI findings; χ^2^/Fisher exact for association with injection response.	Groin pain: SN 100%/SP 7% Snapping hip: SN 24%/SP 82% Pain with resisted SLR: SN 62%/SP 25% Weakness with resisted SLR: SN 15%/SP 71% US bursal distension: SN 67%/SP 35% US tendinosis: SN 63%/SP 32% MRI bursal distension: SN 64%/SP 45% MRI tendon thickening: SN 55%/SP 60% MRI signal heterogeneity: SN 52%/SP 65%
Johnstone et al. (2026) [30]	Retrospective diagnostic accuracy study	105 patients/135 injections (74.8% F); mean age 41.2 ± 15.4 yr (15.6–76.8). 36% post hip arthroscopy, 6.7% post revision arthroscopy, 5.2% post THA. Reference-positive: 77.8% (105/135).	PE: absolute and relative seated hip flexion (SHF) weakness (MRC), pain with SHF, absolute and relative tenderness to palpation (TTP) of the iliopsoas. MRI: any iliopsoas tendon abnormality.	Ultrasound-guided anaesthetic iliopsoas-bursa injection. Positive response: ≥50% pain relief on VAS, immediately or at first follow-up.	SN, SP, PPV, and NPV for each PE manoeuvre and for MRI; logistic regression with subgroup interaction terms.	Absolute SHF weakness: SN 96.2%/PPV 77.1% Relative SHF weakness: SN 82.2%/SP 24.1%/PPV 79.0% Pain with SHF: SN 76.1%/SP 22.2%/PPV 76.9% Absolute TTP iliopsoas: SN 92.6%/PPV 74.6% Relative TTP iliopsoas: SN 86.6%/SP 18.5%/PPV 76.3% MRI abnormality (in 18.1% of scans): SN 19.2%/SP 85.0%

Abbreviations. AUC, area under the receiver operating characteristic curve; ER, external rotation; F, Female; FADIR, flexion–adduction–internal rotation; HEC, hip extension–external rotation–flexion–ceiling (test); HEER, hip extension–external rotation; MCID, minimal clinically important difference; MRC, Medical Research Council; MRI, magnetic resonance imaging; NPRS, Numeric Pain Rating Scale; NPV, negative predictive value; PE, physical examination; PPV, positive predictive value; ROC, receiver operating characteristic; SHF, seated hip flexion; SLR, straight leg raise; SN, sensitivity; SP, specificity; THA, total hip arthroplasty; TTP, tenderness to palpation; US, ultrasound; VAS, visual analog scale.

**Table 3:** (**a**) Conservative treatment of iliopsoas-related groin pain (physiotherapy and injections) in native hips and after total hip arthroplasty. (**b**) Arthroscopic iliopsoas procedures in native hips. (**c**) Endoscopic iliopsoas procedures in native hips. (**d**) Open iliopsoas surgery in native hips. (**e**) Treatment of iliopsoas impingement after total hip arthroplasty (THA).

a.						
Study (Year)	Design	Participants	Indication	Intervention	Follow-Up	Key Outcomes and Conclusions
Johnston et al. (1999) [31]	Retrospective case series	9 patients (8 F); mean age 35.6 ± 12.7 yr	Iliopsoas syndrome (bursitis): anterior hip pain with activity, tenderness to palpation of the femoral triangle, no evidence of hernia, and unremarkable radiologic findings	Home-based progressive rotator strengthening, gluteal re-education and stretching of hip flexors, quadriceps, piriformis, and hamstrings	13.2 ± 9.8 mo	7/9 (77%) returned to full activity; 5/9 (55%) improved by ≥2 activity levels. Conservative rehab effective in most patients.
Laible et al. (2013) [32]	Retrospective case series	49 dancers (43 F); mean age 24.6 (14–49) yr	Iliopsoas syndrome with positive iliopsoas test (pain and/or weakness with resisted hip flexion in external rotation)	NSAIDs, activity modification and physical therapy (iliopsoas stretches, progressive strengthening, pelvic mobilization, antilordotic exercises, long-axis distraction, posterior femoral glide)	Not reported	All patients responded well; none required injection or surgery.
Ruffing et al. (2018) [33]	Retrospective case series	4 patients (3 F); mean age 13.8 (13–15) yr	Acute avulsion fracture of the lesser trochanter	Non-operative: analgesia and partial weight-bearing for 6 weeks	4.9 yr (3.5–6.2)	All returned to competitive sport; HHS 100; ischiofemoral impingement tests negative.
Adler et al. (2005) [21]	Retrospective case series	39 patients; group A: 11 post-THA (7 F; mean age 67); group B: 28 (18 F; mean age 35)	Iliopsoas tendinitis/bursitis (idiopathic or post-THA) refractory to NSAIDs. Groin pain aggravated by hip flexion/SLR	Sonography-guided iliopsoas bursal/peritendinous injection of anaesthetic and corticosteroid	13.5 mo (1–26)	27/39 willing to repeat injection. Post-THA group: 9/10 maintained ≥ 50% relief at 1 yr; non-THA group: 10 of 28 did not experience immediate or delayed relief. 8 of 18 (44%) others achieved sustained relief at 1 yr.
Agten et al. (2015) [34]	Prospective cohort	39 of 112 (27 F); mean age 45 ± 17 yr; 14/39 with THA	Suspected iliopsoas tendinopathy in individuals with or without THA	Fluoroscopy-guided iliopsoas bursa injection (anterolateral approach; anaesthetic and corticosteroid)	1 mo	PGIC clinically improved in 18/37 (49%), unchanged in 13 (35%) and worse in 6 (16%); NRS pain 5.9 ± 2.1 to 3.5 ± 2.5 at 1 mo (*p* ≤ 0.001). No difference with or without THA.
Han et al. (2019) [35]	Prospective cohort	178 patients (154 F); mean age 20.9 ± 7.5 yr (IA hip pathology) and 20.5 ± 7.8 yr (without IA hip pathology)	Clinically diagnosed iliopsoas tendinopathy with or without intra-articular hip pathology	Ultrasound-guided iliopsoas peritendinous corticosteroid injection	6 wk	Significant improvement in HOOS pain, ADL, sport, QoL (all *p* ≤ 0.011). Effect smaller in hips with intra-articular pathology.
Blankenbaker et al. (2006) [36]	Retrospective cohort	40 patients (25 F); mean age 36 (15–72) yr	Clinical diagnosis of snapping hip with painful iliopsoas tendon	Ultrasound-guided injection (anesthetic with or without corticosteroid); arthroscopic release in those with diagnostic relief	4 mo (2–10)	16/18 (89%) bursal corticosteroid injections gave sustained relief; 12/12 patients undergoing later arthroscopic release after positive injection had good outcome. Diagnostic injection predicted surgical success.
Zhu et al. (2020) [37]	Retrospective cohort	45 patients (19 F); mean age 44.5 (18–75) yr	Iliopsoas tendinopathy with associated low-back pain (other hip pathology excluded)	Ultrasound-guided peritendinous injection of anesthetic and corticosteroid	11 mo (10–13)	Low-back pain NRS 7.7 ± 1.3 to 0.8 ± 0.7 at final follow-up; HHS 43.0 ± 16.8 to 98.2 ± 2.6 (*p* < 0.001). No complications. Effective in chronic iliopsoas-related low-back pain.
**b.**						
**Study (year)**	**Design**	**Participants**	**Indication**	**Intervention**	**Follow-up**	**Key outcomes and conclusions**
Maldonado et al. (2018) [38]	Retrospective comparative cohort	307 IFL vs. 354 controls; mean age 27.8 ± 11.4/34.1 ± 12.5 yr	Internal snapping hip on physical examination	Hip arthroscopy with or without iliopsoas fractional lengthening (IFL) at the joint line	IFL: 42.5 ± 18.1 mo; Control: 43.9 ± 19.6 mo	No differences in PROs between IFL and non-IFL groups; PASS 77.6% vs. 75.3% (*p* = 0.57); MCID 75.7% vs. 74.0% (*p* = 0.71). IFL did not adversely affect outcomes.
Jimenez et al. (2022) [39]	Retrospective comparative cohort	105 athletes IFL vs. 52 matched controls; mean age 23/27 yr	Painful internal snapping in competitive athletes with FAI	Hip arthroscopy with intra-bursal IFL at the iliopectineal groove	67 mo	All PROs improved (*p* < 0.001); MCID 82–85% (mHHS, NAHS, HOS-SSS); 89.5% returned to sport. Comparable to controls without IFL.
Perets et al. (2018) [40]	Retrospective comparative cohort	60 athletes IFL (48 F; mean age 19.5 ± 3.9 yr, range 16–34) vs. 41 controls matched on age, sex, and BMI	Painful internal snapping on examination in competitive athletes with FAI	Hip arthroscopy with IFL at the iliopectineal groove	IFL: 49.1 ± 19.1 mo (24–84)	All PROs improved (*p* < 0.001); snapping resolved in 55/60 (91.7%); 39 (65%) returned to sport; mean satisfaction 7.9. No differences vs. control group.
Maldonado et al. (2021) [41]	Retrospective comparative cohort	29 IFL vs. 29 matched controls (all F; borderline acetabular dysplasia); mean age 32.5 ± 11.7 yr (14–58)	Painful internal snapping with cam-FAI and borderline acetabular dysplasia	Femoroplasty + labral repair + IFL + capsular plication (FLIP procedure)	IFL: 48.2 ± 19.0 mo (24–97)	All PROs and VAS improved (*p* < 0.0001); MCID comparable to controls; 7 (9.5%) required revision surgery.
Fabricant et al. (2012) [42]	Retrospective cohort	67 patients (60 F); normal version ≤ 25°: n = 48, mean age 24.4 ± 8.8 yr; high version > 25°: n = 19, mean age 23.0 ± 7.6 yr	Symptomatic coxa saltans confirmed by US-guided diagnostic injection	Arthroscopic transcapsular psoas lengthening with concomitant cam/pincer treatment	1 yr (6–24 mo)	Excessive anteversion (>25°) associated with worse HOS-SSS (26.6 vs. 50.0; *p* = 0.013) and mHHS (76.9 vs. 86.1; *p* = 0.031). High femoral version is a risk factor for poor outcome.
Contreras et al. (2010) [43]	Prospective pilot study	7 patients; mean age 33.6 yr (23–45)	Snapping iliopsoas syndrome	Arthroscopic transcapsular release of the iliopsoas tendon	3, 6, 12, and 24 mo	All snapping resolved; VAS 7.7 (6–10) to 2.4 (0–8) at 24 mo; mHHS 56.1 (13.2–84.7) to 87.9 (49.5–100) at 2 yr (*p* < 0.02). No complications or muscle weakness.
Hwang et al. (2015) [44]	Retrospective cohort	25 patients (5 F); mean age 32 yr (17–53)	Painful internal snapping with concomitant FAI (n = 19) or isolated labral tear (n = 6)	Arthroscopic iliopsoas tendon release with labral repair/debridement and cam/pincer resection	Up to 24 mo	Snapping resolved in 24/25 at 2 yr; HHS 65 (46–86) to 84 (67–98) (*p* < 0.001); 88% good/excellent outcome; HOS-ADL 66 to 87; HOS-SSS 60 to 82; VAS 6 to 2.
Hartigan et al. (2018) [45]	Retrospective cohort	32 patients (30 F); mean age 25 yr	Painful snapping with acetabular dysplasia (LCEA < 25°, mean 21.6°)	Arthroscopic central-compartment IFL with capsular plication and FAI treatment	3 mo–5 yr	All PROs improved (mHHS 68.7 to 83.5; HOS-ADL 64.9 to 86.6; HOS-SSS 71.6 to 86.7; NAHS 52.6 to 75.8; VAS 5.6 to 1.9; all *p* < 0.001). Mean satisfaction 8/10.
El Bitar et al. (2014) [46]	Retrospective case series	55 patients (38 F); mean age 28.2 ± 10.5 yr (15–52)	Internal snapping in patients undergoing hip arthroscopy for labral tear and/or FAI without arthritis (Tönnis 0–1)	Arthroscopic iliopsoas fractional lengthening (IFL)	2.3 ± 0.3 yr (2–3)	All PROs improved (*p* < 0.001): mHHS 62.3 ± 16.4 to 80.5 ± 18.3; NAHS 57.6 ± 20.6 to 80.2 ± 19.2; HOS-ADL 60.9 ± 21.4 to 81.8 ± 20.6; HOS-SSS 43.4 ± 24.6 to 70.0 ± 26.7. 45/55 (82%) reported snapping resolution and good/excellent satisfaction.
Meghpara et al. (2020) [47]	Retrospective cohort	+IPI –PIS –IFL: 38 hips (21 F; mean age 38.2 ± 13.1 yr); +IPI +PIS +IFL: 87 hips (58 F; mean age 31.8 ± 11.9 yr)	Iliopsoas-impingement labral lesion (3-o’clock inflammation) with or without painful internal snapping	Primary hip arthroscopy for FAI and IPI ± IFL (only when painful snapping present)	≈31 mo (24–66)	Both groups improved comparably (all *p* < 0.01). +IPI –PIS –IFL: mHHS 63.9 ± 13.1 to 86.1 ± 15.3; NAHS 65.1 ± 15.8 to 83.0 ± 18.0; HOS-SSS 40.0 ± 19.3 to 78.1 ± 20.7; VAS 5.5 ± 2.3 to 2.2 ± 2.3. +IPI +PIS +IFL: mHHS 63.6 ± 16.2 to 86.1 ± 16.5; NAHS 62.5 ± 18.2 to 84.7 ± 17.3; HOS-SSS 41.4 ± 23.3 to 76.5 ± 25.5; VAS 4.9 ± 2.4 to 2.2 ± 2.6. IFL not required when painful snapping is absent.
Perets et al. (2019) [39]	Retrospective cohort	57 patients (50 F); mean age 26.5 ± 10.7 yr (15–52)	Painful internal snapping with FAI/labral tear	Hip arthroscopy ± IFL at the level of the joint	69.3 mo (60–92)	All PROs improved (mHHS 64.3 to 84.9; NAHS 61.7 to 85.2; HOS-SSS 47.0 to 75.0; VAS 6.5 to 2.2); satisfaction 8.1/10. Snapping resolved in 80.7%. 17.5% required secondary arthroscopy; 5.3% conversion to THA.
Anderson et al. (2008) [48]	Retrospective case series	15 athletes (11 F); 5 competitive (mean age 17.4 yr, 15–21) and 10 recreational (mean age 38 yr, 17–62); mean symptom duration 22 mo	Painful snapping hip relieved by anaesthetic injection into the psoas bursa	Arthroscopic iliopsoas tendon release	17 mo (12–33)	mHHS competitive 41 (20–62) to 97 (92–100) at 12 mo; recreational 44 (14–72) to 96 (92–100) at 12 mo. Effective in both populations.
Nelson et al. (2014) [49]	Retrospective case series	30 patients (24 F); mean age 35 yr (15–57)	Tight psoas tendon impinging on a torn or inflamed labrum at arthroscopy	Arthroscopic iliopsoas tenotomy at the level of the labrum	3, 6, 12 and 24 mo	mHHS 43 (26–59) to 88 (65–100) at 24 mo. Three patients required additional injections; 2 needed further release at the lesser trochanter.
Matsuda et al. (2021) [50]	Retrospective cohort	Tenotomy: 16 (63% F; mean age 38 ± 17 yr); iliopsoas no-tenotomy: 76 (59% F; mean age 35 ± 14 yr); control: 1301 (69% F; mean age 35 ± 13 yr)	FAI ± coxa saltans internus with anteroinferior labral lesion and positive psoas injection	Hip arthroscopy for FAI ± transcapsular/endoscopic iliopsoas tenotomy	2.05 yr (1.8–2.2)	iHOT-12 tenotomy 35 ± 15 to 57 ± 28 (25% reached normal function) vs. no-tenotomy 36 ± 19 to 69 ± 32 (49%) vs. control 35 ± 18 to 73 ± 24 (41%). Tenotomy associated with inferior functional recovery.
Brandenburg et al. (2016) [51]	Retrospective cohort	Release: 18 patients (13 F; mean age 31.9 ± 9.2 yr); control: 18 patients (13 F; mean age 34.1 ± 9.9 yr)	Symptomatic internal snapping hip	Hip arthroscopy for FAI/chondrolabral damage ± iliopsoas release at the level of the joint	21 mo (16–30)	Release: 25% smaller iliopsoas muscle volume (288 ± 98 vs. 384 ± 113 cm^3^; *p* < 0.001) and 19% lower seated hip flexion strength (13 ± 5 vs. 17 ± 6 kg; *p* < 0.001). No supine strength difference. Structural sequelae after release.
Patel et al. (2020) [52]	Retrospective review	44 patients (46 hips; 35 F)	Symptomatic internal snapping hip	Hip arthroscopy with arthroscopic iliopsoas release	582 ± 461 d (71–2076)	Iliopsoas cross-sectional area decreased by 191 mm^2^ at AIIS (*p* < 0.001) and 587 ± 315 mm^2^ at L5 (*p* < 0.0001). Postoperative mHHS 51.0 ± 19.6; HOS-ADL 55.7 ± 19.8; HOS-sports 41.7 ± 28.5. Quantifiable muscle atrophy after release.
Mardones et al. (2016) [53]	Retrospective case series	15 patients (11 F); mean age 33.5 ± 10.7 yr (18–49)	FAI with iliopsoas tendinopathy (palpation pain, pain on resisted hip flexion, imaging-confirmed)	Arthroscopic FAI surgery with transcapsular iliopsoas tendon release	4 yr	mHHS 74.7 ± 17 (39.6–93.4) to 95.8 ± 8.4 (69–100); VAIL 53 ± 15 (30–78) to 85 ± 14 (57–100) (*p* < 0.05); VAS median 5.5 (3–7) to 0 (0–5) (*p* < 0.001). Two recurrences at 1 yr managed conservatively. No major complications.
**c.**						
**Study (year)**	**Design**	**Participants**	**Indication**	**Intervention**	**Follow-up**	**Key outcomes and conclusions**
Flanum et al. (2007) [54]	Retrospective case series	6 patients (5 F); mean age 46.6 yr	Painful snapping iliopsoas tendon	Endoscopic iliopsoas tendon release	Up to 12 mo	HHS 58 pre-operatively to 90 at 6 mo and 96 at 12 mo.
Ilizaliturri et al. (2015) [55]	Retrospective cohort	28 patients (16 F; mean age 29.3 ± 14.4 yr, 16–65); single-tendon group n = 23, multiple-tendon group n = 5	Internal snapping hip syndrome	Endoscopic transcapsular release at the central compartment	30.6 mo (12–53)	WOMAC single-tendon group 39 (95% CI 26–55) to 73.6 (95% CI 68–80); multiple-tendon group 47.2 (95% CI 44–58) to 77.9 (95% CI 68–83). Comparable outcomes regardless of tendon anatomy.
Ilizaliturri et al. (2005) [56]	Consecutive case series	6 patients (7 hips, all F); mean age 38.5 yr (26–44)	Internal snapping hip with audible snap on hip extension from a flexed position	Endoscopic release of the iliopsoas tendon at the lesser trochanter	21.4 mo (10–27)	WOMAC 82.5 ± 5.0 to 91.0 ± 1.3. First description of endoscopic release at the lesser trochanter.
Loppini et al. (2026) [57]	Monocentric retrospective cohort	20 patients (17 F); mean age 42.4 yr (24–55); 18/20 (90%) had concomitant intra-articular procedures	Painful snapping hip refractory to ≥6 mo of conservative treatment ± concomitant intra-articular pathology	Endoscopic transcapsular partial iliopsoas tenotomy via radiofrequency device	10.6 yr (10–13)	mHHS 63.7 ± 5.9 to 88.3 ± 6.2; HOOS 71.5 ± 7.2 to 86.7 ± 5.9 at last follow-up; 95% satisfied/very satisfied; persistent hip flexion weakness in 3/20 (15%). Durable long-term benefit.
**d.**						
**Study (year)**	**Design**	**Participants**	**Indication**	**Intervention**	**Follow-up**	**Key outcomes and conclusions**
Garala et al. (2014) [58]	Retrospective case–control	36 patients (28 F); mean age 49.4 ± 11.9 yr (31–80)	Painful snapping hip on extension from flexion–abduction–external rotation	Open psoas tenotomy via medial Ludloff approach (n = 15) vs. image-guided steroid injection (n = 23)	Tenotomy: 49 mo (13–144); injection: 77 mo (14–160)	NAHS higher after injection (86; 95% CI 75–98) than after tenotomy (68; 95% CI 57–78) (*p* < 0.05); pain reduced more after injection (4.2 to 1.8; 95% CI 1.2–2.3) than after tenotomy (4.1 to 2.9) (*p* < 0.01). 89% would recommend injection vs. 74% tenotomy.
Hoskins et al. (2004) [59]	Retrospective review	80 patients/92 hips (50 F); mean age 27.3 yr (15–52)	Painful snapping iliopsoas tendon recalcitrant to conservative treatment	Open iliopsoas fractional lengthening (four partial tenotomies) via an inguinal approach	5.4 yr	89% satisfaction; 40 complications in 32 patients (9 recurrent snapping, 11 outright failures, 8 sensory deficits, 6 persistent pain, 3 flexion weakness). High morbidity profile.
Dobbs et al. (2002) [60]	Retrospective review	9 patients/11 hips (7 F); mean age 15.2 ± 1.0 yr	Persistent painful snapping in adolescents reproduced on extension of the flexed and abducted hip	Open fractional lengthening at the musculotendinous junction via a modified iliofemoral approach	4 yr (2–10)	1 recurrence; all returned to baseline activity; no detectable strength loss; 2 transient anterolateral thigh sensory disturbances.
Gruen et al. (2002) [61]	Retrospective cohort	11 patients (7 F); mean age 31.5 yr (16–54)	Painful internal snapping reproduced on active extension of the hip from a flexed position	Open tendon lengthening through a true ilioinguinal approach	3 yr (10 mo–6 yr)	Snapping resolved in all; 91% satisfied with pain relief; 73% returned to sport (45% to previous level); residual flexion weakness in 1/11; 82% would repeat surgery.
**e.**						
**Study (year)**	**Design**	**Participants**	**Indication**	**Intervention**	**Follow-up**	**Key outcomes and conclusions**
Batailler et al. (2017) [62]	Retrospective cohort	46 patients (33 F); mean age 66 ± 12 yr (44–85)	Iliopsoas impingement after THA due to anterior cup prominence (83%; mean 9.9 mm) and/or cup malposition (76%)	Acetabular cup revision	21 ± 16 mo (6–72)	OHS 19.7 ± 7 (7–35) to 43 ± 6 (25–48) (*p* < 0.001); 85% satisfied; 41/46 pain-free; complications in 3 (6.5%); recurrent groin pain in 4 (8.7%).
Bell et al. (2019) [63]	Retrospective cohort	60 patients (36 F); mean age 62 ± 9.7 yr	Iliopsoas impingement after THA	Endoscopic iliopsoas tenotomy	5.5 mo	93.3% pain resolution; HOS-ADL 57.5 ± 18.8 (10.9–89.3) to 71.6 ± 26.1 (14.1–100) (*p* = 0.005); HOS-SSS 37.3 ± 24.0 to 58.1 ± 33.2 (*p* = 0.002). One postoperative hematoma.
Benad et al. (2015) [64]	Retrospective case series	5 patients (3 F); mean age 64 yr (53–75)	Iliopsoas impingement after THA refractory to extra-articular corticosteroid injections	Ambulatory bursectomy with Vicryl™ mesh on the anterior hip capsule via a Hueter approach	13.6 mo (9–29)	Mean improvement: OHS +15 (10–19), Merle d’Aubigné +2.5 (1–5) and HHS +18 (10–29). One infected hematoma.
Di Benedetto et al. (2019) [65]	Retrospective case series	13 patients (4 F; 11 THA, 1 endoprosthesis, 1 resurfacing); mean age 65 yr (47–82)	Iliopsoas tendinopathy due to impingement after hip arthroplasty	Arthroscopic tendon release at the impingement zone on the anterior cup rim	10 mo	HHS 66.8 (48.9–81.8) to 85 (80–95); VAS 3.6 (2–6) to 1 (0–3); MRC 3.6 (3–4) to 4.7 (3–5); hip flexion ROM 95° (80–100°) to 105° (90–120°).
Dora et al. (2007) [22]	Retrospective cohort	Conservative: 8 patients (5 F; mean age 57 yr); tenotomy: 6 (3 F; mean age 73 yr); revision: 16 (10 F; mean age 62 yr)	Iliopsoas impingement after THA with malpositioned or oversized acetabular component, pain on SLR and resisted hip flexion	Conservative care, iliopsoas tenotomy or acetabular cup revision (multi-arm)	Conservative 41 mo; tenotomy 36 mo; revision 40 mo (24–65)	HHS gain greatest after revision (60 [48,49,50,51,52,53,54,55,56,57,58,59,60,61,62,63,64,65,66,67,68,69,70,71,72,73,74,75,76,77,78,79,80,81,82,83,84,85] to 82 [55,56,57,58,59,60,61,62,63,64,65,66,67,68,69,70,71,72,73,74,75,76,77,78,79,80,81,82,83,84,85,86,87,88,89,90,91,92,93,94,95]); tenotomy 59 (56–67) to 73 (67–89); conservative essentially unchanged (57 [50,51,52,53,54,55,56,57,58,59,60,61,62,63,64,65,66,67,68] to 58 [42,43,44,45,46,47,48,49,50,51,52,53,54,55,56,57,58,59,60,61,62,63,64,65,66,67,68]). Revision favoured when the cup is malpositioned.
Buller et al. (2021) [8]	Retrospective cohort	32 patients (25 F); mean age 60.3 ± 11.6 yr (34–80)	Iliopsoas impingement defined as groin pain ≥ 6 wk postoperatively, aggravated by resisted hip flexion or sit-to-stand	Stepwise: home exercises → physical therapy → corticosteroid injection → arthroscopic release ± cup revision	51.9 ± 20.4 mo (24–95)	Treatment success (>80% symptom reduction): 16% (5/32) home exercises, 31% (8/26) PT, 25% (3/12) injection, 67% (4/6) release, 50% (1/2) revision. Surgical options outperform conservative care.
Nunley et al. (2009) [66]	Retrospective case series	27 patients (15 F); mean age 59 yr (31–84)	Iliopsoas tendonitis after THA	Selective iliopsoas-bursa anaesthetic + corticosteroid injection	44.6 mo (25–68)	HHS 61 to 82 (*p* < 0.001); VAS 6.4 (3–10) to 2.9 (0–8) (*p* < 0.001). 22% required additional surgical procedure.
Valenzuela & O’Donnell (2021) [67]	Retrospective case series	35 patients (20 F; mean age 62 ± 10.3 yr, 40–84); lesser trochanter (LT) group n = 21, acetabular-rim (AR) group n = 14	Iliopsoas impingement after THA confirmed by US-guided injection	Endoscopic tenotomy at the lesser trochanter (LT) vs. the acetabular rim (AR)	LT: 49.1 ± 20.5 mo; AR: 42.4 ± 12.3 mo	VAS LT 5.3 ± 1.1 to 1.8 ± 1.8 (*p* < 0.001); AR 5.8 ± 1.3 to 2.6 ± 2.2. mHHS 89.0 ± 10.3 (LT) vs. 81.1 ± 12.4 (AR); NAHS 86.0 ± 11.1 (LT) vs. 78.9 ± 10.1 (AR); satisfaction 3.2 ± 0.9 vs. 2.7 ± 1.2 (all n.s.). LT may give marginally better function.
Viamont-Guerra et al. (2021) [68]	Retrospective case series	48 patients/50 hips (32 F); mean age 60.8 ± 10.5 yr (36–80)	Iliopsoas tendinopathy after THA refractory to ≥6 mo conservative care	Endoscopic iliopsoas tenotomy between the lesser trochanter and the psoas valley	31.3 ± 15.3 mo (12–72)	mHHS 57.7 ± 11.5 (31.9–81.4) to 83.2 ± 16.9 (41.0–100) and OHS 29.4 ± 11.0 (4.0–50.0) to 50.5 ± 8.4 (28.0–60.0) (both *p* < 0.001); 87% satisfied. 4/50 required cup ± stem revision.
Finsterwald et al. (2024) [69]	Retrospective case series	36 patients (25 F); mean age 62 ± 12 yr (27–83)	Iliopsoas impingement after THA, confirmed by positive psoas sheath injection	Endoscopic iliopsoas tendon release at the lesser trochanter	24 mo	HHS 59 ± 19.5 (18.7–94.6) to 82.9 ± 11.9 (61.6–100) (*p* < 0.001); VAS 5.7 ± 1.7 (2.9–9) to 2.1 ± 2.1 (0–7) (*p* < 0.001); SANE 54.3 ± 22.6 (1–91) to 75.7 ± 17.1 (40–100) (*p* = 0.013). Peak isometric hip flexion strength 20% lower on the operated side (*p* < 0.001).
Guicherd et al. (2017) [70]	Prospective case series	64 patients (40 F); mean age 56.3 yr (33–78)	Impingement between the acetabular component and the iliopsoas tendon	Endoscopic tenotomy at the lesser trochanter (n = 57) or arthroscopic tenotomy at the psoas notch (n = 7)	8 mo	OHS 21.8 to 40 (*p* < 0.001); pain with seated hip flexion 92% to 19% and pain on SLR 95% to 21% (both *p* < 0.001). 94% pain alleviation; 87% satisfaction.
Bonano et al. (2023) [71]	Retrospective case series	24 patients (16 F); mean age 69 yr (IQR 63–74)	Iliopsoas tendinopathy due to impingement after THA, with significant pain relief from ≥1 image-guided iliopsoas injection	Endoscopic iliopsoas lengthening at the lesser trochanter	7.6 mo (1–28)	mHHS median 57 (IQR 43–60) to 75 (IQR 66–92) (*p* < 0.001); iHOT-12 median 71 (IQR 48–80); VAS median 3 (IQR 0.8–5); 71% satisfied/very satisfied. Two deep infections requiring two-stage THA revision.
Portet et al. (2024) [72]	Retrospective cohort	29 patients (14 F); mean age 62.6 ± 12.2 yr	Iliopsoas impingement after THA, positive infiltrative test	Endoscopic iliopsoas tenotomy at the lesser trochanter	3.6 ± 0.8 yr	mHHS gain 36.7 ± 23.3, OHS gain 19.8 ± 12.7 and VAS reduction 5.5 ± 3.3 at last follow-up (all *p* < 0.001). 37% reduction in seated and 60% reduction in supine hip flexion strength (both *p* < 0.001).
Jerosch et al. (2013) [73]	Retrospective case series	35 patients; age range 58–82 yr	Iliopsoas impingement after THA with positive local-anaesthetic test and anterior-prominent acetabular component	Arthroscopic release of the iliopsoas tendon at the proven lesion	3.6 yr (6 mo–12 yr)	32/35 reported pre-operative pain disappeared 1 d postoperatively. All patients had transient first-week weakness which recovered by follow-up.
Tassinari et al. (2021) [74]	Retrospective case series	16 patients (11 F); mean age 57.8 ± 11.1 yr (36–75)	Iliopsoas impingement after THA confirmed by US-guided peritendinous injection	Arthroscopic iliopsoas release (Wettstein technique)	27 ± 20.1 mo (6–48)	WOMAC 36.1 ± 8.6 (28–44) to 83.4 ± 9.5 at final follow-up (*p* < 0.05); 81% complete pain relief; 88% achieved MRC 5 at final follow-up. One required cup revision.
Van Riet et al. (2011) [75]	Retrospective case series	9 patients; mean age 51 yr (24–81)	Iliopsoas impingement after THA	Arthroscopic psoas release	11 mo (4–20)	HOOS 41.7 ± 19.3 (10.6–73.8) to 58.6 ± 21.1 (32.5–100) (*p* = 0.10); MRC 2.7 ± 0.5 (2–3) to 5 (*p* < 0.0001).
Nikou et al. (2023) [76]	Retrospective case series	12 patients/13 hips (8 F); mean age 64.4 ± 15.1 yr	Iliopsoas impingement after THA	Arthroscopic iliopsoas tenotomy at the anteromedial aspect of the acetabular cup	49.8 ± 25 mo	HAGOS subscales (pain, sport, ADL, QoL) improved (*p* < 0.05); iHOT-12 24.9 to 34.5 (n.s.); 10/12 (83%) satisfied.
Simon et al. (2024) [77]	Retrospective cohort	58 patients/60 hips (39 F); mean age 64.1 ± 11.2 yr	Iliopsoas tendinitis after THA	Arthroscopic IFL proximal to the lesser trochanter (≈2 cm) with muscular preservation	39.3 mo (12–106)	Anterior groin pain better in 75%; mHHS 73.9 ± 19.4; SANE 71.9 ± 21.9; Tegner 2.5 ± 1.7 to 2.9 ± 1.4 (*p* = 0.025); VAS at rest 1.3 ± 2.4. Revision THA in 3 (5.2%).
Nazal et al. (2019) [78]	Retrospective case series	10 patients (7 F); mean age 60 ± 8.4 yr (51–80)	Iliopsoas tendinopathy after THA	Capsular release around the impingement zone with proximal iliopsoas recession (no full tenotomy)	81.6 ± 16.8 mo (47–110)	VAS 8.5 ± 1.7 (5–10) to 0.8 ± 2.2 (0–7) (*p* = 0.006); flexion ROM 91.5° ± 11.8° to 99° ± 6.6° (*p* = 0.018); 6/10 improved SLR strength. Revision THA in 2 (20%).
Williams & Ashworth (2019) [79]	Prospective case series	13 patients (11 F); mean age 52.8 ± 13.7 yr (29–83)	Psoas impingement after THA confirmed by US-guided steroid injection of the psoas bursa	Arthroscopic extra-articular psoas tenotomy at the lesser trochanter	2 yr (0.7–7)	8/13 (62%) pain-free; 9/13 negative postoperative FABER; mean subjective flexion weakness 20% (0–30%).
Martinot et al. (2025) [80]	Retrospective cohort	55 hips (37 F); mean age 58 yr (35–81); 12 hips had previously failed iliopsoas tenotomy (n = 8) or capsule-thickening plasty (n = 4)	Iliopsoas impingement after THA, including failed prior tenotomy or capsule plasty	Acetabular cup revision via Moore’s posterolateral approach	3 yr (2–10)	OHS (Dawson 12–60 scale; lower = better) median 40 (IQR 37–45) at baseline to 18 (IQR 15–28) at follow-up; median gain 18 (IQR 15–27; *p* < 0.001); MCID achieved in 48/55 (87%); PASS (≤21/60) in 33/55 (67%). PMA median 17 (IQR 15–18); MRC median 4.5 (IQR 4–5). 40/55 (73%) satisfied or very satisfied. Major complications in 6 (10.9%).
Nogier et al. (2024) [5]	Retrospective case series	7 patients (5 F); mean age 55.9 ± 5.5 yr (50–65)	Anterior acetabular wall defect with associated iliopsoas tendinopathy after THA	Anterior acetabular wall reconstruction with an autologous biconvex iliac crest graft and press-fit revision cup	60.3 ± 5.6 mo (52–70)	MRC 3.9 ± 0.7 to 4.6 ± 0.9; FJS 77.5 ± 32.8 (13–100); OHS 40.3 ± 12.8 (16–48); VAS 0.2 ± 0.4 at rest (0–1) and 2.3 ± 2.6 during activities (0–6); satisfaction 8.3 ± 2.3 (5–10). Three of four imaged grafts osseointegrated.
O’Sullivan et al. (2007) [81]	Retrospective case series	15 patients (11 F); mean age 55.5 yr (33–75)	Iliopsoas tendonitis after THA with painful active hip flexion	Open release of the iliopsoas tendon from the lesser trochanter	36.4 mo (5–63)	HHS 58 (44–70) to 91 (78–95); complete resolution of groin pain in 11/15 (69%); satisfaction 9.3/10.
Moreta et al. (2021) [82]	Retrospective case series	12 patients (6 F); mean age 59.1 yr (40–72)	Iliopsoas impingement after THA, positive diagnostic iliopsoas sheath injection	Outside-in arthroscopic iliopsoas release at the edge of the cup	45 mo (24–96)	HHS 58.8 ± 10.1 (37–76) to 86.1 ± 12 (59–98) (*p* = 0.001); VAS 6.2 ± 1.0 (4–8) to 1.1 ± 1.4 (0–5) (*p* < 0.001).
Zimmerer et al. (2022) [83]	Retrospective cohort	20 patients (10 F); mean age 59 yr (52–78)	Mechanical iliopsoas irritation after cementless THA with minimal anterior cup prominence (<8 mm)	Arthroscopic iliopsoas release at the impingement zone	7 ± 3.8 yr (2.6–11.7)	mHHS 31.2 ± 9.8 (17.6–47.3) to 82.0 ± 9.8 (46.2–100) (*p* = 0.001); NPRS 8.5 ± 1.2 (7–10) to 2.5 ± 1.8 (0–6) (*p* = 0.001); UCLA activity 4.0 ± 2.7 (0–7) to 6.5 ± 1.8 (3–9) (n.s.).
Homma et al. (2024) [84]	Phase 1 prospective observational cohort	3 patients (2 F); aged 65, 66 and 73 yr	Iliopsoas impingement after THA with positive xylocaine test	Locoregional autologous platelet-rich plasma (PRP) injection at the impingement site under fluoroscopic and US guidance	12 wk	≥20% pain reduction in all patients; 2/3 had ≥20% FJS-12 improvement; WOMAC unchanged. One patient developed diverticulitis at 2 mo.
Erard et al. (2024) [85]	Retrospective case series	49 hips in 47 patients (31 F); mean age 60.7 ± 10.6 yr (36–80)	Iliopsoas tendinopathy after THA, diagnosed by groin pain on active hip flexion with exclusion of alternative causes	Endoscopic tenotomy between the lesser trochanter and the psoas valley, anterior to the capsule	68.8 ± 9.3 mo	OHS 32.5 ± 10.2 (13–55) to 55.4 ± 6.0 (31–60) (MCID 92.5%; PASS 97.5%); mHHS 58.0 ± 12.0 (32–81) to 88.6 ± 13.9 (45–100); 92.5% satisfied/very satisfied. Seven hips required revision.
Yun et al. (2021) [86]	Retrospective case series	8 patients (2 F); mean age 62 yr (44–74)	Iliopsoas impingement after THA	Acetabular cup revision via a direct anterior approach with partial iliopsoas tenotomy at the lesser trochanter	39.6 mo (12–108)	Postoperative HOOS-JR 75 (32–100). Combined approach feasible in selected revision cases.
Gédouin & Huten (2012) [87]	Retrospective case series	10 patients (5 F); mean age 58 yr (45–80)	Iliopsoas tendinopathy secondary to THA	Endoscopic tenotomy at the lesser trochanter	20 mo (12–60)	WOMAC 34 (24–46) to 84 (60–95); PMA 13.1 (11–15) to 16.9 (15–18); 9/10 satisfied or very satisfied.
Filanti et al. (2016) [88]	Retrospective cohort	11 patients (5 F); mean age 57 yr (29–77)	Iliopsoas impingement after THA	Arthroscopic debridement of adhesions ± transcapsular iliopsoas tenotomy (Wettstein technique)	24 mo	HHS 44.1 ± 7.3 (32–56) to 75.7 ± 15.5 (50–91) (debridement) and 83.3 (61–91) (debridement + release, n = 7); MRC 3.3 (3–4) to 4.5 (3–5).
Weintraub et al. (2023) [89]	Retrospective cohort	42 patients (23 F); mean age 61 yr (43–85)	Iliopsoas bursitis after THA	Ultrasound-guided iliopsoas bursal injection	41 mo (12–125)	HHS 71.6 (50–96) to 86.2 (56–100) (*p* < 0.001). Effective non-operative option.
Martinot et al. (2024) [90]	Retrospective case series	14 patients (10 F); median age 57.5 yr (IQR 51–64.5)	Iliopsoas impingement after THA	Capsule-thickening plasty with folded Vicryl™ mesh via a Hueter approach	4 yr (1–9)	OHS gain median 7 (IQR 0–13; *p* = 0.004); MRC median 5 (IQR 5–5); 7/14 (50%) satisfied or very satisfied; 1 major and 2 minor complications. Variable medium-term outcomes.
Lambrey et al. (2023) [91]	Prospective cohort	51 patients (33 F); mean age 69 ± 10.2 yr (42–89)	Iliopsoas impingement after THA	Endoscopic or arthroscopic iliopsoas tenotomy	60 mo	OHS 21.8 to 40.7 ± 7.7 (12–48) (*p* < 0.001); VAS 2.1 ± 2.3 (0–10); satisfaction 8.0 ± 2.1 (1–10). Durable benefit at 5 yr.
Chalmers et al. (2017) [92]	Retrospective cohort	Conservative: 20 patients (13 F; mean age 69 yr); tenotomy: 8 (mean age 62 yr); revision: 21 (mean age 62 yr)	Iliopsoas impingement after primary THA	Conservative care with injections, open iliopsoas tenotomy, or acetabular revision (multi-arm)	Conservative: 4.2 yr (1–12); operative: 3.5 yr (2–8)	Resolution of groin pain in 50% conservative vs. 76% operative. HHS gain greatest after revision (53 to 79) and tenotomy (53 to 86); both *p* < 0.001.
Carbonell-Rosell et al. (2022) [93]	Retrospective cohort	25 patients (14 F); mean age 67.4 ± 13.4 yr	Iliopsoas impingement after THA confirmed by US-guided injection of corticosteroid and local anaesthetic	Conservative care (analgesics, physiotherapy, infiltrations) vs. endoscopic iliopsoas tenotomy	46.2 ± 20.9 mo	Conservative: 12/25 (48%) improved (HHS 80.2 ± 5.0; VAS 3.0 ± 1.8). Tenotomy: 13/25 (52%) operated, of whom 8/13 (62%) improved (HHS 87.8 ± 12.8; VAS 1.5 ± 1.7). Axial cup overhang ≥ 10 mm predicts poor tenotomy outcome.
Howell et al. (2021) [94]	Retrospective review	24 patients; mean age 54 yr	Clinical and radiological diagnosis of iliopsoas pathology after THA	US-guided steroid + physiotherapy, open tenotomy, or cup revision	Not reported	Steroid: complete resolution 14/23 (61%); 44% of responders later relapsed and required surgery. Open tenotomy: complete resolution in 4/7 (57%); cup revision: complete resolution.
Holliday et al. (2025) [95]	Retrospective cohort	52 patients (36 F; mean age 63.9 ± 11.7 yr); arthroscopic IFL: n = 36 (mean age 66.8 ± 9.3 yr); open tenotomy: n = 16 (mean age 57.4 ± 13.6 yr)	Iliopsoas tendinopathy after THA, confirmed by US-guided diagnostic injection	Open iliopsoas tenotomy vs. arthroscopic iliopsoas fractional lengthening (IFL)	68.7 mo (24–204)	Arthroscopic IFL trended toward higher satisfaction (7.9 vs. 6.0), greater groin-pain improvement (86 vs. 45%), and lower revision-THA rate (8.3 vs. 25%). VAS at rest 1.1 ± 2.2 vs. 3.3 ± 3.2 favoured IFL (*p* = 0.009); mHHS 77.0 ± 19.0 vs. 69.2 ± 26.9 (*p* = 0.073).
Pate et al. (2025) [96]	Retrospective case series	15 patients (7 F); mean age 58 ± 11 yr	Anterior iliopsoas impingement after THA	Arthroscopic iliopsoas lengthening at the level of impingement after failed conservative management	3.8 yr (1.3–6.8)	Median VAS 8 to 0 (*p* < 0.001); 12/15 (80%) pain-free at final follow-up. No revisions, reoperations, or infections.

**Legend.** Studies are grouped by treatment setting and ordered chronologically within each group. Numeric outcome data are presented as mean ± SD, median (interquartile range), or as a range where reported by the original authors; p values are included where reported. Where two or more groups are reported, the results are presented in the order in which the interventions appear in the column. **Abbreviations:** ADLs, activities of daily living; AIIS, anterior inferior iliac spine; AR, acetabular rim; CT, computed tomography; DAA, direct anterior approach; ER, external rotation; FABER, flexion–abduction–external rotation; FAI, femoroacetabular impingement; FJS-12, Forgotten Joint Score-12; FLIP, femoroplasty + labral repair + iliopsoas fractional lengthening + plication; HAGOS, Copenhagen Hip and Groin Outcome Score; HHS, Harris Hip Score; HOOS, Hip disability and Osteoarthritis Outcome Score; HOOS-JR, HOOS Joint Replacement; HOS-ADL/SSS, Hip Outcome Score—ADL/Sport-Specific Subscale; iHOT-12, International Hip Outcome Tool-12; IFL, iliopsoas fractional lengthening; IPI, iliopsoas impingement; IQR, interquartile range; JHEQ, Japanese Hip Evaluation Questionnaire; LCEA, lateral center-edge angle; LT, lesser trochanter; MCID, minimal clinically important difference; mHHS, modified HHS; MRC, Medical Research Council; MRI, magnetic resonance imaging; n.r./NR, not reported; NAHS, Non-Arthritic Hip Score; NPRS, Numeric Pain Rating Scale; NRS, Numeric Rating Scale; NSAID, non-steroidal anti-inflammatory drug; OHS, Oxford Hip Score; PASS, Patient Acceptable Symptom State; PGIC, Patient Global Impression of Change; PIS, painful internal snapping; PMA, Postel Merle d’Aubigné; PRO, patient-reported outcome; PRP, platelet-rich plasma; QoL, quality of life; ROM, range of motion; RTS, return to sport; SANE, Single-Assessment Numeric Evaluation; SLR, straight-leg raise; THA, total hip arthroplasty; UCLA, University of California, Los Angeles activity score; US, ultrasound; VAIL, Vail Hip Score; VAS, visual analog scale; VR-12 P/M, Veterans RAND-12 Physical/Mental; W, women; WOMAC, Western Ontario and McMaster Universities Osteoarthritis Index.

**Table 4:** Risk of bias and applicability assessment of the included diagnostic accuracy studies (QUADAS-3 tool).

Study	Risk of Bias				Applicability Concerns			Overall Risk of Bias
	Participants	Index Test	Target Condition	Analysis	Participants	Index Test	Target Condition	
Vandeputte et al. (2025) [29]	Low	High	High	Low	Low	Low	Low	High
Haskel et al. (2021) [20]	High	High	High	Low	Low	Low	Low	High
Johnstone et al. (2026) [30]	High	High	High	High	High	Low	Low	High

Legend. Risk of bias and applicability were assessed by two reviewers using the QUADAS-3 tool [27].

**Table 5:** Methodological quality of the included treatment studies (MINORS—Methodological Index for Non-Randomized Studies).

Study (Year)	1	2	3	4	5	6	7	8	9 *	10 *	11 *	12 *	Total
***Conservative treatment—physiotherapy and injections***													
Johnston et al. (1999) [31]	2	1	1	1	0	2	1	0	–	–	–	–	**8/16**
Laible et al. (2013) [32]	2	2	1	2	0	2	0	0	–	–	–	–	**9/16**
Ruffing et al. (2018) [33]	2	2	1	2	1	2	2	0	–	–	–	–	**12/16**
Adler et al. (2005) [21]	2	0	1	1	0	2	2	0	1	2	0	1	**12/24**
Agten et al. (2015) [34]	2	1	2	2	0	2	1	0	–	–	–	–	**10/16**
Han et al. (2019) [35]	2	2	2	2	0	2	0	0	–	–	–	–	**10/16**
Blankenbaker et al. (2006) [36]	2	2	1	2	1	1	2	0	1	2	0	2	**16/24**
Zhu et al. (2020) [37]	2	2	1	2	0	2	1	0	–	–	–	–	**10/16**
***Arthroscopic treatment—native hip***													
Maldonado et al. (2018) [38]	2	2	2	2	0	2	1	2	1	2	1	2	**19/24**
Jimenez et al. (2022) [39]	2	2	2	2	0	2	1	2	2	2	1	2	**20/24**
Perets et al. (2018) [40]	2	1	2	2	0	2	1	2	2	2	2	2	**20/24**
Maldonado et al. (2021) [41]	2	2	2	2	0	2	1	2	2	2	2	2	**21/24**
Fabricant et al. (2012) [42]	2	2	2	2	0	2	2	1	2	2	1	2	**20/24**
Contreras et al. (2010) [43]	2	0	2	2	0	2	2	0	–	–	–	–	**10/16**
Hwang et al. (2015) [44]	2	2	2	2	0	2	2	0	–	–	–	–	**12/16**
Hartigan et al. (2018) [45]	2	2	2	2	0	2	1	0	–	–	–	–	**11/16**
El Bitar et al. (2014) [46]	2	2	2	2	0	2	1	0	–	–	–	–	**11/16**
Meghpara et al. (2020) [47]	2	2	2	2	0	2	1	2	2	2	1	2	**20/24**
Perets et al. (2018) [40]	2	2	2	2	0	2	1	2	2	2	1	2	**20/24**
Anderson et al. (2008) [48]	2	2	2	2	0	2	1	0	–	–	–	–	**11/16**
Nelson et al. (2014) [49]	2	2	2	2	0	2	1	0	–	–	–	–	**11/16**
Matsuda et al. (2021) [50]	2	2	2	2	0	2	1	0	2	1	2	2	**18/24**
Brandenburg et al. (2016) [51]	2	1	2	2	0	2	1	2	2	2	2	2	**20/24**
Patel et al. (2020) [52]	2	1	1	2	0	2	1	0	–	–	–	–	**9/16**
Mardones et al. (2016) [53]	2	1	1	2	0	2	1	0	–	–	–	–	**9/16**
***Endoscopic treatment—native hip***													
Flanum et al. (2007) [54]	2	1	2	2	0	2	1	0	–	–	–	–	**10/16**
Ilizaliturri et al. (2015) [55]	2	2	2	2	2	2	2	0	–	–	–	–	**14/16**
Ilizaliturri et al. (2005) [56]	2	2	1	2	0	2	2	0	–	–	–	–	**11/16**
Loppini et al. (2026) [57]	2	2	2	2	0	2	2	1	–	–	–	–	**13/16**
***Open surgery—native hip***													
Garala et al. (2014) [58]	2	1	1	2	0	2	1	0	1	2	0	2	**14/24**
Hoskins et al. (2004) [59]	2	2	1	2	0	2	1	0	–	–	–	–	**10/16**
Dobbs et al. (2002) [60]	2	0	1	2	0	2	2	0	–	–	–	–	**9/16**
Gruen et al. (2002) [61]	2	1	1	0	0	2	2	0	–	–	–	–	**8/16**
***Treatment after total hip arthroplasty***													
Batailler et al. (2017) [62]	2	2	0	2	0	2	2	0	–	–	–	–	**10/16**
Bell et al. (2019) [63]	2	2	1	2	1	1	2	0	1	2	1	2	**17/24**
Benad et al. (2015) [64]	0	0	0	2	0	1	2	0	–	–	–	–	**5/16**
Di Benedetto et al. (2019) [65]	2	2	2	2	0	1	1	0	–	–	–	–	**10/16**
Dora et al. (2007) [22]	2	2	2	2	0	2	2	0	1	2	1	0	**16/24**
Buller et al. (2021) [8]	2	2	1	1	0	2	1	0	2	2	1	2	**16/24**
Nunley et al. (2009) [66]	2	1	2	2	0	2	1	0	–	–	–	–	**10/16**
Valenzuela & O’Donnell (2021) [67]	2	0	2	2	0	2	2	0	1	1	2	2	**16/24**
Viamont-Guerra et al. (2021) [68]	2	2	0	2	0	2	1	0	–	–	–	–	**9/16**
Finsterwald et al. (2024) [69]	2	2	2	2	0	2	1	2	–	–	–	–	**13/16**
Guicherd et al. (2017) [70]	2	2	2	2	0	2	2	0	–	–	–	–	**12/16**
Bonano et al. (2023) [71]	2	2	1	2	0	1	1	0	–	–	–	–	**9/16**
Portet et al. (2024) [72]	2	2	1	2	0	2	1	0	–	–	–	–	**10/16**
Jerosch et al. (2013) [73]	1	0	0	1	0	2	2	0	–	–	–	–	**6/16**
Tassinari et al. (2021) [74]	2	2	1	2	0	2	2	0	–	–	–	–	**11/16**
Van Riet et al. (2011) [75]	2	1	2	2	0	2	2	0	–	–	–	–	**11/16**
Nikou et al. (2023) [76]	2	2	2	2	1	2	2	0	–	–	–	–	**13/16**
Simon et al. (2024) [77]	2	2	1	2	0	2	1	0	–	–	–	–	**10/16**
Nazal et al. (2019) [78]	2	2	2	2	0	2	2	0	–	–	–	–	**12/16**
Williams & Ashworth (2019) [79]	2	2	2	1	0	2	2	0	–	–	–	–	**11/16**
Martinot et al. (2025) [80]	2	2	1	2	2	2	2	0	–	–	–	–	**13/16**
Nogier et al. (2024) [5]	2	2	1	2	1	2	1	0	–	–	–	–	**11/16**
O’Sullivan et al. (2007) [81]	2	2	0	2	0	2	2	0	–	–	–	–	**10/16**
Moreta et al. (2021) [82]	2	2	0	2	2	2	2	0	–	–	–	–	**12/16**
Zimmerer et al. (2022) [83]	2	2	0	2	0	2	2	0	–	–	–	–	**10/16**
Homma et al. (2024) [84]	2	0	2	2	0	2	2	0	–	–	–	–	**10/16**
Erard et al. (2024) [85]	2	2	2	2	0	2	2	0	–	–	–	–	**12/16**
Yun et al. (2021) [86]	2	2	0	2	0	2	2	0	–	–	–	–	**10/16**
Gédouin & Huten (2012) [87]	2	1	0	2	0	2	2	0	–	–	–	–	**9/16**
Filanti et al. (2016) [88]	2	2	0	2	0	2	2	0	–	–	–	–	**10/16**
Weintraub et al. (2023) [89]	2	2	0	2	0	2	1	0	1	2	2	2	**16/24**
Martinot et al. (2024) [90]	2	2	0	2	2	2	2	0	–	–	–	–	**12/16**
Lambrey et al. (2023) [91]	2	2	2	2	0	2	2	0	–	–	–	–	**12/16**
Chalmers et al. (2017) [92]	2	2	1	2	0	2	1	0	1	2	0	2	**15/24**
Carbonell-Rosell et al. (2022) [93]	2	2	1	2	2	2	2	0	1	2	2	2	**20/24**
Howell et al. (2021) [94]	2	2	2	2	0	2	2	0	1	2	0	2	**17/24**
Holliday et al. (2025) [95]	2	2	1	2	0	2	1	0	2	1	1	2	**16/24**
Pate et al. (2025) [96]	2	1	1	2	0	2	2	0	–	–	–	–	**10/16**

* Applicable only to comparative studies.

## Data Availability

No new data were created or analyzed in this study. Data sharing is not applicable to this article.

## Supplementary Materials

The following supporting information can be downloaded at https://www.mdpi.com/article/10.3390/jcm15155912/s1, Table S1: PRISMA 2020 Checklist.

## Abbreviations

The following abbreviations are used in this manuscript:

ADLs	activities of daily living
AIIS	anterior inferior iliac spine
AR	acetabular rim
AUC	area under the receiver operating characteristic curve
BMI	body mass index
CI	confidence interval
CT	computed tomography
DAA	direct anterior approach
ER	external rotation
FABER	flexion–abduction–external rotation
FADIR	flexion–adduction–internal rotation
FAI	femoroacetabular impingement
FJS-12	Forgotten Joint Score-12
FJVDP	Frans-Jozef Vandeputte (author initialism)
FLIP	femoroplasty + labral repair + iliopsoas fractional lengthening + capsular plication
HAGOS	Copenhagen Hip and Groin Outcome Score
HEC	hip–external rotation–flexion–ceiling (test)
HEER	hip extension–external rotation
HHS	Harris Hip Score
HOOS	Hip disability and Osteoarthritis Outcome Score
HOOS-JR	HOOS Joint Replacement
HOS-ADL	Hip Outcome Score—Activities of Daily Living subscale
HOS-SSS	Hip Outcome Score—Sport-Specific Subscale
IA	intra-articular
IFL	iliopsoas fractional lengthening
iHOT-12	International Hip Outcome Tool-12
IPI	iliopsoas impingement
IQR	interquartile range
IRGP	iliopsoas-related groin pain
JHEQ	Japanese Hip Evaluation Questionnaire
LCEA	lateral center-edge angle
LR	likelihood ratio
LT	lesser trochanter
MCID	minimal clinically important difference
MeSH	Medical Subject Headings
mHHS	modified Harris Hip Score
MINORS	Methodological Index for Non-Randomized Studies
MRC	Medical Research Council
MRI	magnetic resonance imaging
NAHS	Non-Arthritic Hip Score
NPV	negative predictive value
NPRS	Numeric Pain Rating Scale
NRS	Numeric Rating Scale
NSAID	non-steroidal anti-inflammatory drug
OHS	Oxford Hip Score
PASS	Patient Acceptable Symptom State
PE	physical examination
PGIC	Patient Global Impression of Change
PICOS	population, intervention, comparator, outcome, study design
PIS	painful internal snapping
PMA	Postel Merle d’Aubigné score
PPV	positive predictive value
PRISMA	Preferred Reporting Items for Systematic Reviews and Meta-Analyses
PRO	patient-reported outcome
PROM	patient-reported outcome measure
PRP	platelet-rich plasma
PROSPERO	International Prospective Register of Systematic Reviews
QUADAS-3	Quality Assessment of Diagnostic Accuracy Studies-3
QoL	quality of life
ROC	receiver operating characteristic
ROM	range of motion
RTS	return to sport
SANE	single-assessment numeric evaluation
SD	standard deviation
SHF	seated hip flexion
SLR	straight-leg raise
SN	sensitivity
SP	specificity
THA	total hip arthroplasty
TTP	tenderness to palpation
UCLA	University of California Los Angeles activity score
US	ultrasound
VAIL	Vail Hip Score
VAS	visual analog scale
VR-12 P/M	Veterans RAND-12 Physical/Mental composite score
WOMAC	Western Ontario and McMaster Universities Osteoarthritis Index

## Appendix A

### Appendix A.1. Search Strategy for Diagnostic Accuracy (PubMed, 540 Results)

(“iliopsoas”[Title/Abstract] OR “iliopsoas muscle”[Title/Abstract] OR “iliopsoas-related”[Title/Abstract] OR “groin pain”[Title/Abstract] OR “hip flexor”[Title/Abstract] OR muscle, psoas[MeSH] OR “psoas” [Title/Abstract] OR “anterior hip pain”[Title/Abstract]) AND (“diagnosis”[MeSH] OR “diagnosis”[Title/Abstract] OR “diagnostic test”[Title/Abstract] OR “clinical test*”[Title/Abstract] OR “clinical sign*”[Title/Abstract] OR “physical examination”[Title/Abstract] OR “index test*”[Title/Abstract] OR “diagnostic criteri*”[Title/Abstract] OR “test*”[Title/Abstract]) AND (“diagnostic imaging”[MeSH Terms] OR “magnetic resonance imaging”[MeSH Terms] OR “ultrasonography”[MeSH Terms] OR “diagnostic imaging”[Title/Abstract] OR “magnetic resonance imaging”[Title/Abstract] OR “ultrasonography”[Title/Abstract] OR MRI[Title/Abstract] OR ultrasound[Title/Abstract] OR “surg* findings”[Title/Abstract] OR “surg* confirmation”[Title/Abstract] OR “imaging”[Title/Abstract] OR “sonograph*”[Title/Abstract] OR “criterion reference*”[Title/Abstract] OR “reference standard*”[Title/Abstract] OR “arthroscop*”[Title/Abstract] OR “tend* repair”[Title/Abstract] OR tenotomy[MeSH Terms] OR tenotomy[Title/Abstract]) AND (“diagnostic accuracy”[Title/Abstract] OR “sensitivity and specificity”[MeSH] OR “sensitivity”[Title/Abstract] OR “specificity”[Title/Abstract] OR “predictive value of tests”[MeSH] OR “predictive value”[Title/Abstract] OR “true positiv*”[Title/Abstract] OR “false positiv*”[Title/Abstract] OR “true negativ*”[Title/Abstract] OR “false negativ*”[Title/Abstract] OR “diagnostic utility”[Title/Abstract] OR “diagnostic propert*”[Title/Abstract] OR validity[Title/Abstract] OR reliability[Title/Abstract] OR predictiv*[Title/Abstract]).

### Appendix A.2. Search Strategy for Treatment Studies (PubMed, 4.208 Results, 3.188 with Filter “Humans”)

(“iliopsoas”[Title/Abstract] OR “iliopsoas muscle”[Title/Abstract] OR “iliopsoas-related”[Title/Abstract] OR “groin pain”[Title/Abstract] OR “hip flexor”[Title/Abstract] OR muscle, psoas[MeSH] OR “psoas” [Title/Abstract] OR “anterior hip pain”[Title/Abstract]) AND (“treatment”[Title/Abstract] OR “therapy”[Title/Abstract] OR “rehabilitation”[MeSH] OR “exercise therapy”[MeSH] OR “physical therapy modalities”[MeSH] OR “surgery”[Title/Abstract] OR “injection”[Title/Abstract] OR “infiltration”[Title/Abstract] OR “shock wave therapy”[Title/Abstract] OR “exercise therapy”[Title/Abstract] OR “medication”[Title/Abstract] OR “passive modalities”[Title/Abstract] OR “manual therapy”[Title/Abstract] OR “strength*”[Title/Abstract] OR “physiotherapy”[Title/Abstract] OR “surgery”[Title/Abstract] OR “arthroscop*”[Title/Abstract] OR “tenotom*”[Title/Abstract] OR “corticosteroid injection”[Title/Abstract]) AND (“pain”[MeSH] OR “outcome”[Title/Abstract] OR “treatment outcome”[MeSH] OR “function*”[Title/Abstract] OR “recovery”[Title/Abstract] OR “self-reported pain”[Title/Abstract] OR “self-reported function”[Title/Abstract] OR “performance-based outcomes”[Title/Abstract] OR “pain”[Title/Abstract] OR “disability”[Title/Abstract]).
