# Supplementary material for: Clinical Diagnosis and Treatment of Iliopsoas-Related Groin Pain: A Systematic Review
Source: J Clin Med. 2026 Jul 29;15(15):5912. doi: 10.3390/jcm15155912 (PMC13466767; doi:10.3390/jcm15155912)
Supplement: Supplementary file 1 [file jcm-15-05912-s001.zip › jcm-4379827-supplementary.pdf]

Table S1. PRISMA 2020 Checklist

Section and Topic	Item #	Checklist item	Location where item is reported
**TITLE**			
Title	1	Identify the report as a systematic review.	Page 1 - "Clinical diagnosis and treatment of iliopsoas-related groin pain: a systematic review".
**ABSTRACT**			
Abstract	2	See the PRISMA 2020 for Abstracts checklist.	Page 1–2 – Abstract: Background/Objectives, Methods, Results, Conclusions, Registration (PROSPERO CRD42025644593).
**INTRODUCTION**			
Rationale	3	Describe the rationale for the review in the context of existing knowledge.	Page 2 – Introduction, paragraphs 1–4.
Objectives	4	Provide an explicit statement of the objective(s) or question(s) the review addresses.	Page 2 – Introduction, final paragraph.
**METHODS**			
Eligibility criteria	5	Specify the inclusion and exclusion criteria for the review and how studies were grouped for the syntheses.	Page 3 – Section 2.1 "Eligibility criteria" + Table 1 (PICOS framework for diagnostic accuracy and treatment effectiveness, with explicit exclusion criteria).
Information sources	6	Specify all databases, registers, websites, organisations, reference lists and other sources searched or consulted to identify studies. Specify the date when each source was last searched or consulted.	Page 3–4 – Section 2.2 "Information sources": PubMed, Embase, Web of Science (last search 13 March 2026); reference lists of included studies and reviews were hand-screened.
Search strategy	7	Present the full search strategies for all databases, registers and websites, including any filters and limits used.	Page 3–4 – Section 2.3 "Search strategy"; full search strategies in Appendix A (pages 39–40).
Selection process	8	Specify the methods used to decide whether a study met the inclusion criteria of the review, including how many reviewers screened each record and each report retrieved, whether they worked independently, and if applicable, details of automation tools used in the process.	Page 4 – Section 2.4 "Selection process": two reviewers (FJVDP, AS) screened independently in Rayyan; disagreements resolved by a third reviewer (SR); no automation tools used.
Data collection process	9	Specify the methods used to collect data from reports, including how many reviewers collected data from each report, whether they worked independently, any processes for obtaining or confirming data from study investigators, and if applicable, details of automation tools used in the process.	Page 4 – Section 2.5 "Data collection process": data extracted by AS, checked by FJVDP; authors contacted to clarify missing information; no automation tools used.
Data items	10a	List and define all outcomes for which data were sought. Specify whether all results that were compatible with each outcome domain in each study were sought (e.g. for all measures, time	Page 4 – Section 2.6.1 "Outcomes": sensitivity, specificity, likelihood ratios and raw 2×2 data for diagnostic studies; self-reported pain (VAS, NRS), PROMs (HHS, HOOS, OHS, iHOT) and performance-based outcomes for treatment studies; all reported time-points were extracted.

Table S1. PRISMA 2020 Checklist

Section and Topic	Item #	Checklist item	Location where item is reported
		points, analyses), and if not, the methods used to decide which results to collect.	
	10b	List and define all other variables for which data were sought (e.g. participant and intervention characteristics, funding sources). Describe any assumptions made about any missing or unclear information.	Page 4 – Section 2.6.2 "Other variables": study design, sample size, patient characteristics, underlying aetiology of IRGP (idiopathic, post-arthroscopy, post-THA), intervention characteristics, follow-up duration and complications.
Study risk of bias assessment	11	Specify the methods used to assess risk of bias in the included studies, including details of the tool(s) used, how many reviewers assessed each study and whether they worked independently, and if applicable, details of automation tools used in the process.	Page 4–5 – Section 2.7 "Study risk of bias assessment": QUADAS-3 (Whiting et al., 2026) for diagnostic studies; MINORS (Slim et al., 2003) for treatment studies; two reviewers independently, disagreements resolved by consensus.
Effect measures	12	Specify for each outcome the effect measure(s) (e.g. risk ratio, mean difference) used in the synthesis or presentation of results.	Page 4–5 – Section 2.8 "Effect measures": sensitivity, specificity and likelihood ratios for diagnostic outcomes; mean differences and changes from baseline for treatment outcomes. No standardised effect sizes were calculated due to heterogeneity.
Synthesis methods	13a	Describe the processes used to decide which studies were eligible for each synthesis (e.g. tabulating the study intervention characteristics and comparing against the planned groups for each synthesis (item #5)).	Page 5 – Section 2.9 "Synthesis methods": studies grouped by research question (diagnosis vs. treatment), intervention type and clinical context (native hip / post-arthroscopy / post-THA).
	13b	Describe any methods required to prepare the data for presentation or synthesis, such as handling of missing summary statistics, or data conversions.	Page 5 – Section 2.9 "Synthesis methods": data tabulated to summarise study characteristics, outcomes and risk of bias (Tables 2–5, pages 8–32). No data conversions or handling of missing summary statistics was required.
	13c	Describe any methods used to tabulate or visually display results of individual studies and syntheses.	Page 5 – Section 2.9 "Synthesis methods" + Tables 2–5 (pages 8–32); PRISMA 2020 flow diagrams presented as Figures 1 and 2 (page 6).
	13d	Describe any methods used to synthesize results and provide a rationale for the choice(s). If meta-analysis was performed, describe the model(s), method(s) to identify the presence and extent of statistical heterogeneity, and software package(s) used.	Page 5 – Section 2.9 "Synthesis methods": narrative synthesis performed because heterogeneity in populations, interventions, outcome measures and study designs precluded meaningful quantitative pooling. No meta-analysis was conducted.
	13e	Describe any methods used to explore possible causes of heterogeneity among study results (e.g. subgroup analysis, meta-regression).	Page 5 – Section 2.9 "Synthesis methods": subgroup analyses performed descriptively based on aetiology (idiopathic, post-arthroscopy, post-THA) and treatment category.
	13f	Describe any sensitivity analyses conducted to assess robustness of the synthesized results.	Page 5 – Section 2.9 "Synthesis methods": "No sensitivity analyses were conducted."
Reporting bias	14	Describe any methods used to assess risk of bias	Not formally assessed (pages 5, 29–32) – due to heterogeneity and absence of a meta-analysis, no funnel plot or

Table S1. PRISMA 2020 Checklist

Section and Topic	Item #	Checklist item	Location where item is reported
assessment		due to missing results in a synthesis (arising from reporting biases).	reporting-bias test was performed.
Certainty assessment	15	Describe any methods used to assess certainty (or confidence) in the body of evidence for an outcome.	Not formally performed; evidence strength discussed narratively in the Discussion (pages 33–37).
**RESULTS**			
Study selection	16a	Describe the results of the search and selection process, from the number of records identified in the search to the number of studies included in the review, ideally using a flow diagram.	Page 5–6 – Section 3.1 "Study selection and design" + Figures 1 and 2 (PRISMA 2020 flow diagrams, page 6).
	16b	Cite studies that might appear to meet the inclusion criteria, but which were excluded, and explain why they were excluded.	NA
Study characteristics	17	Cite each included study and present its characteristics.	Pages 8–22 – Tables 2 (diagnostic studies, page 8) and 3 (treatment studies, pages 10–21) cite each included study and present its characteristics.
Risk of bias in studies	18	Present assessments of risk of bias for each included study.	Pages 29–32 – Table 4 (QUADAS-3 for diagnostic studies, page 29) and Table 5 (MINORS for treatment studies, pages 30–32) present the per-study risk-of-bias assessment.
Results of individual studies	19	For all outcomes, present, for each study: (a) summary statistics for each group (where appropriate) and (b) an effect estimate and its precision (e.g. confidence/credible interval), ideally using structured tables or plots.	Pages 8–22 – Tables 2 and 3 present summary statistics for each individual study (sensitivity / specificity / AUC / pain and functional outcomes pre→post, follow-up). Numerical claims repeated narratively in sections 3.3–3.4 (pages 22–29).
Results of syntheses	20a	For each synthesis, briefly summarise the characteristics and risk of bias among contributing studies.	Page 29 – Section 3.5 "Risk of bias within studies" briefly summarises the characteristics and risk of bias of contributing studies for each synthesis category.
	20b	Present results of all statistical syntheses conducted. If meta-analysis was done, present for each the summary estimate and its precision (e.g. confidence/credible interval) and measures of statistical heterogeneity. If comparing groups, describe the direction of the effect.	Not applicable – only narrative synthesis was performed; no statistical pooling. See pages 22–29.
	20c	Present results of all investigations of possible causes of heterogeneity among study results.	Pages 24–29 – Section 3.4 "Treatment effects by intervention category and etiology" presents subgroup-stratified findings (idiopathic / athletic / post-THA; matched-comparative cohort studies vs. single-arm series).
	20d	Present results of all sensitivity analyses conducted to assess the robustness of the synthesized results.	Not applicable – no sensitivity analyses were conducted (stated in Methods, page 5).
Reporting biases	21	Present assessments of risk of bias due to missing results (arising from reporting biases) for each synthesis assessed.	Pages 29–32 – A formal assessment of risk of bias due to missing results could not be performed because of heterogeneity and the absence of a quantitative synthesis.

PRISMA

Table S1. PRISMA 2020 Checklist

Section and Topic	Item #	Checklist item	Location where item is reported
Certainty of evidence	22	Present assessments of certainty (or confidence) in the body of evidence for each outcome assessed.	Not formally performed; evidence strength discussed narratively in the Discussion (pages 33–37).
**DISCUSSION**			
Discussion	23a	Provide a general interpretation of the results in the context of other evidence.	Page 33 – Discussion, "Summary of findings": interprets the diagnostic and treatment findings in the context of existing literature (Anderson 2016, Tramer 2024, Weir 2015).
	23b	Discuss any limitations of the evidence included in the review.	Page 36 – Discussion, "Limitations and future directions": heterogeneity of study designs, interventions, outcome measures and diagnostic criteria; predominance of non-randomised studies; absence of validated diagnostic reference standard.
	23c	Discuss any limitations of the review processes used.	Page 36 – Discussion, "Limitations and future directions": limitations of the review process including predominance of retrospective designs, potential publication bias and reliance on injection-based reference standard.
	23d	Discuss implications of the results for practice, policy, and future research.	Pages 36–37 – Discussion, "Limitations and future directions" and closing paragraphs of "Treatment strategies" and "Prognosis and patient selection".
**OTHER INFORMATION**			
Registration and protocol	24a	Provide registration information for the review, including register name and registration number, or state that the review was not registered.	Page 1 (Abstract) and page 3 (Section 2, first paragraph) – PROSPERO CRD42025644593.
	24b	Indicate where the review protocol can be accessed, or state that a protocol was not prepared.	Page 3 – Section 2, first paragraph: "prospectively registered in the PROSPERO database (CRD42025644593)".
	24c	Describe and explain any amendments to information provided at registration or in the protocol.	NA
Support	25	Describe sources of financial or non-financial support for the review, and the role of the funders or sponsors in the review.	Page 37 – Funding section.
Competing interests	26	Declare any competing interests of review authors.	Page 37 – Conflicts of Interest section.
Availability of data, code and other materials	27	Report which of the following are publicly available and where they can be found: template data collection forms; data extracted from included studies; data used for all analyses; analytic code; any other materials used in the review.	NA

From: Page MJ, McKenzie JE, Bossuyt PM, Boutron I, Hoffmann TC, Mulrow CD, et al. The PRISMA 2020 statement: an updated guideline for reporting systematic reviews. BMJ 2021;372:n71. doi: 10.1136/bmj.n71.
This work is licensed under CC BY 4.0. To view a copy of this license, visit https://creativecommons.org/licenses/by/4.0/
